# Allelic Expression Imbalance in the Human Retinal Transcriptome and Potential Impact on Inherited Retinal Diseases

**DOI:** 10.3390/genes8100283

**Published:** 2017-10-20

**Authors:** Pablo Llavona, Michele Pinelli, Margherita Mutarelli, Veer Singh Marwah, Simone Schimpf-Linzenbold, Sebastian Thaler, Efdal Yoeruek, Jan Vetter, Susanne Kohl, Bernd Wissinger

**Affiliations:** 1 Institute for Ophthalmic Research, Centre for Ophthalmology, 72076 Tuebingen, Germany; Simone.Schimpf-Linzenbold@humangenetik-tuebingen.de (S.S.-L.); Susanne.Kohl@med.uni-tuebingen.de (S.K.); Bernd.Wissinger@med.uni-tuebingen.de (B.W.); 2 Telethon Institute of Genetics and Medicine, 80078 Pozzuoli, Italy; m.pinelli@tigem.it (M.P.); mutarelli@tigem.it (M.M.); veer.marwah@helsinki.fi (V.S.M.); 3 Institute of Biotechnology, University of Helsinki, 00014 Helsinki, Finland; 4 Center for Ophthalmology, 72076 Tuebingen, Germany; sebastian.thaler@med.uni-tuebingen.de; 5 Augenklinik Mülheim, 45468 Mülheim an der Ruhr, Germany; efdal.yoeruek@evkmh.de; 6 Universitäts-Augenklinik, 55131 Mainz, Germany; jan.vetter@gmx.de

**Keywords:** Inherited retinal diseases, penetrance, allelic expression imbalance, retina, expressivity

## Abstract

Inherited retinal diseases (IRDs) are often associated with variable clinical expressivity (VE) and incomplete penetrance (IP). Underlying mechanisms may include environmental, epigenetic, and genetic factors. *Cis*-acting expression quantitative trait loci (*cis*-eQTLs) can be implicated in the regulation of genes by favoring or hampering the expression of one allele over the other. Thus, the presence of such loci elicits allelic expression imbalance (AEI) that can be traced by massive parallel sequencing techniques. In this study, we performed an AEI analysis on RNA-sequencing (RNA-seq) data, from 52 healthy retina donors, that identified 194 imbalanced single nucleotide polymorphisms(SNPs) in 67 IRD genes. Focusing on SNPs displaying AEI at a frequency higher than 10%, we found evidence of AEI in several IRD genes regularly associated with IP and VE (*BEST1*, *RP1*, *PROM1*, and *PRPH2*). Based on these SNPs commonly undergoing AEI, we performed pyrosequencing in an independent sample set of 17 healthy retina donors in order to confirm our findings. Indeed, we were able to validate *CDHR1*, *BEST1*, and *PROM1* to be subjected to *cis*-acting regulation. With this work, we aim to shed light on differentially expressed alleles in the human retina transcriptome that, in the context of autosomal dominant IRD cases, could help to explain IP or VE.

## 1. Introduction

Inherited retinal diseases (IRDs) are a group of genetic disorders resulting in retinal dysfunction and/or degeneration which in turn lead to visual impairment and, in some instances, to complete blindness. Until now, almost 200 genes associated with IRD have been identified with all modes of inheritance represented (RetNet database https://sph.uth.edu/retnet/, June 2017) [1]. In addition, IRDs are frequently associated with phenotypic variability in terms of various distinct retinal disease entities and even inter- and intrafamilial phenotypic heterogeneity in subjects carrying the same mutation [2,3]. Such diversity is thought to be due to environmental, epigenetic, and genetic factors. Focusing on this latter group, features like numerous allelic mutations in the same disease-associated gene, a diverse set of genes involved in the same disease, and multigenetic factors provide some molecular answers for the wide range of observed phenotypes. However, several IRD cases have been reported in which a certain mutation results in affected or unaffected mutation carriers, implicating incomplete penetrance (IP) or variable expressivity (VE) [2]. The former would refer to those cases in which a given mutation does not induce the corresponding disease in all mutation carriers, whereas the latter indicates ranges of symptomatology and severity in carriers of the same genetic condition.

In many instances, the cause and underlying molecular mechanism of IP and VE remains elusive. To tackle this issue, the concept of modifier genes has been proposed. In this scenario, the pathology induced by the principle gene defect is worsened or ameliorated at least to some extent by the action of a secondary gene, for instance by altering the expression of the primary gene or through activating a surrogate signaling pathway [4,5,6].

Expression quantitative trait loci (eQTLs) may be alternative mechanisms for IP and VE. Such eQTLs have the ability to interfere with the expression of genes by affecting the levels of transcription and translation. From the molecular point of view, eQTLs are mainly single nucleotide polymorphisms (SNPs) or repetitive elements present within regulatory elements that can be categorized into two groups depending on their location and effect with respect to a given gene [7]. *trans*-eQTLs are situated at distal positions, possibly on a distinct chromosome, equally influencing the expression of both alleles of the target gene, whereas *cis*-eQTLs act locally and differentially in relation to each allele. In particular, *cis*-eQTLs can alter transcription initiation if located within promoters, enhancers, or insulators, or affect mRNA stability—for instance, through regulatory elements located in non-coding regions of transcripts or by inducing differential splicing (sQTL) [8,9,10]. Indeed, the presence of these *cis*-acting regulatory elements entail quantitative transcript alterations in an allele-specific fashion, also known as allelic expression imbalance (AEI) [11,12]. An example illustrating these concepts in the context of IRDs was established some years ago in *PRPF31-*associatedautosomal dominant retinitis pigmentosa (adRP). Asymptomatic carriers of a dominant mutation were shown to have increased levels of mRNA deduced from the wild type allele, thus compensating for the lack of function of the mutant allele [13]. Further investigations in *PRPF31* identified that extra copies of a *cis*-acting minisatellite repeat element (MSR1), adjacent to its core promoter, repressed gene transcription [14]. Furthermore, a putative *trans*-acting factor (*CNOT3*) has been proposed to act in *cis* over *PRPF31* [15]. Needless to say, this phenomenon could also act on autosomal recessive or X-linked cases; however, its impact would be hard to assess since compound heterozygosity or X-chromosome inactivation are respectively characteristic of these cases [16].

Since RNA-sequencing (RNA-seq) has been introduced for genome-wide transcript analysis, an increasing number of studies apply this technology to address AEI [17,18], progressively replacing other technologies such as RT-qPCR (real-time quantitative polymerase chain reaction) and genome wide-SNP (single nucleotide polymorphisms) arrays. The advantage of RNA-seq in comparison to other technologies is the amount of information that is able to be generated and processed as well as the automation of the analysis in combination to a relatively low workload. In general, next generation sequencing and in particular short read applications are not optimally suited for allele quantification, since it tends to give rise to biased results due to mapping errors. Taking this into account, it is necessary to validate AEIs obtained from the analysis of RNA-seq data by an independent method such as pyrosequencing, a well-established method with excellent performance in allele quantification.

Although in recent years the number of publications addressing AEI analysis has thrived, when it comes to the ophthalmic field, there is still need for a better understanding of how genes are frequently controlled by *cis*-regulators in the retina context. To gain insight on this issue, we reanalyzed RNA-seq data from 50 human healthy retina donors from a previous study by performing a bioinformatics AEI analysis [19], after analyzing the very limited data of two RNA-seq generated locally. Follow-up pyrosequencing experiments focusing on the most prominent SNPs gathered in RNA-seq were carried out on an independent sample-set of 17 human healthy eye donors, from which retinal RNA and genomic DNA extracted from the sclera of the same eyecup were available for validation. All in all, here we provide a comprehensive dataset of SNPs that commonly undergo AEI in genes associated with IRD in humans; furthermore, we try to hallmark such genes for researchers and physicians in future investigations and efforts to analyzes and explain IP or VE cases.

## 2. Materials and Methods

### 2.1. Human Eye Collection

We collected 17 post-mortem healthy human eyes from donors of German origin (Table S1) for whom informed consent for research purposes had been obtained with Project No. 105/2009BO1 and date of votum 13.07.2009. Demographic data including sex and birth date were only available for 11/17 subjects (three female, eight male; age 40–85 years at time of death, mean 68 years). Eyecups were dissected and retinae were placed into RNA Stabilization Reagent (RNA later, QIAGEN; Hilden, Germany) to limit RNA degradation with a mean post-mortem interval of 30 h (ranging from 5 to 80 h). Pieces of the sclera were also prepared and frozen at −80°C for later DNA extraction.

### 2.2. DNA and RNA Isolation

DNA was obtained from 30 mg of sclera after overnight treatment with Proteinase K (VWR Peqlab, Darmstadt, Germany) and DNA extraction using a Tissue DNA Mini Kit, PeqGold (VWR Peqlab) according to the manufacturer’s protocols. Total RNA was isolated from whole retina using an RNeasy Mini Kit (Qiagen; Hilden, Germany). RNA preparationwas analyzed on a Bioanalyzer 2100 (Agilent Technologies, Waldbronn, Germany) and RNA integrity number (RIN) was calculated as an estimate of RNA quality (Table S1).

### 2.3. RNA-sequencing

To obtain full view of retinal transcriptome, two retina RNA samples from two independent healthy human eye donors were selected for RNA-seq (Table S1). Libraries were sequenced using paired-end chemistry, according to the standard operating procedures (SOPs) of the Institute of Medical Genetics and Applied Genomics, University of Tuebingen, Germany, and Microarray Facility Tuebingen Services (Tuebingen, Germany). No further details on this process were provided by these companies. Coherent transcripts were assessed by visual inspection using Integrative Genomics Viewer (IGV).

### 2.4. Allelic Expression Imbalance RNA-sequencing Analysis

To conduct the AEI analysis, we used the two initially prepared RNA-seq datasets plus the 50 human RNA-seq retinal samples derived from our previous study (deposited in http://www.ebi.ac.uk/arrayexpress/experiments/E-MTAB-4377/files/) [19]. These latter 50 samples were sequenced on an Illumina HiSeq1000 platform (Illumina Inc., San Diego, CA, USA) with paired-end chemistry.

**Figure 1:**
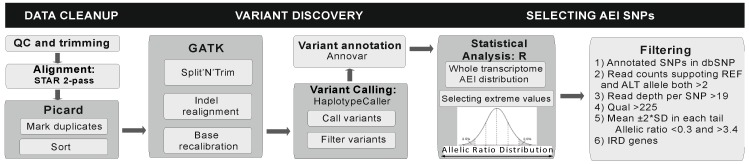
RNA-seq allelic expression imbalance (AEI) analysis workflow. Detailed are all steps contained in the pipeline (see also 2.4. Allelic expression imbalance (AEI) RNA-sequencing analysis). This process is subcategorized into three major blocks: Data cleanup, variant discovery, and selection of AEI single nucleotide polymorphism (SNPs). QC – quality control, GATK - Genome Analysis Toolkit, Indel – insertion/deletion, REF – reference, ALT – alternative, Qual –quality, IRD – inherited retinal disease.

The whole procedure, detailed in Figure 1, was carried out following the GATK (Genome Analysis Toolkit) best practice (https://software.broadinstitute.org/gatk/documentation/article.php?id=3891). In brief, trimmed reads were aligned to the human genome (UCSC (University of California Santa Cruz) genome browser version hg19; https://genome.ucsc.edu/) [20] with two sequential passes of STAR (ultrafast universal RNA-seq aligner) [21]. Duplicate reads and sorting were performed with Picard. The splicing-junction re-alignment, local re-alignment, and base quality recalibration were performed with GATK. The variant calling and variant annotation were carried out with GATK’s Haplotype Caller and Annovar, respectively.

R and RStudio were used to perform the statistical analysis. We selected the single nucleotide variants that had a QUAL larger than 225, coverage of at least 20x, each allele supported by at least two reads, and were also reported in dbSNP (Single Nucleotide Polymorphism database https://www.ncbi.nlm.nih.gov/projects/SNP/) [22]. For each variant, we calculated the log-ratio between alternative and reference read counts. Assuming a normal distribution, the outlier values were defined as those outside the range of mean±2*SD (standard deviation). Subsequently, we selected the variants that had outliers in at least one sample. In addition, we kept also variants that had one outlier sample and at least one moderately imbalanced variant, defined as a linear-ratio outside 0.666 and 1.5 (equivalent to allele percentages in the range of 40–60%).

In a second step, IRD genes selected according to the RetNet database (http://www.sph.uth.tmc.edu/RetNet/) [1], filtered, and their AEI frequency was calculated for each SNP. With those variants displaying AEI frequencies higher than 10%, we estimated the orientation of the allelic ratio to find out whether these SNPs are potentially in linkage disequilibrium (LD) with the causal AEI variant or haplotype. Next, we calculated the overall strength of the imbalance (absolute allele mean ± standard error of the mean (SEM)) by orientating all samples towards the allele with the lesser read counts (A) devided by the allele with most read counts fraction (B). Finally, we estimated kurtosis (and standard error of kurtosis, SEK) to point out those SNPs for which samples’ allelic ratios are clustered around the mean value, suggesting a single molecular reason for AEI.

### 2.5. Single Nucleotide PolymorphismHeterozygosity Confirmation by Sanger Sequencing

Sanger sequencing of the 17 samples of the testing set was performed to confirm SNP heterozygosity out of PCR-amplified genomic scleral DNA prior to pyrosequencing analysis. Primer pairs were designed with Primer-BLAST (https://www.ncbi.nlm.nih.gov/tools/primer-blast/) [23] and checked for potential misalignment with the BLAST-like alignment tool (https://genome.ucsc.edu/cgi-bin/hgBlat) [24] (Table S2). PCR fragments were purified (ExoSAP-IT enzyme cleanup; USB, Cleveland, OH) andsequenced with dye-termination chemistry (Big Dye Terminationchemistry; Applied Biosystems [ABI], Weiterstadt, Germany). Products were separated on a DNA capillary sequencer (3100 Genetic Analyzer; ABI, Weiterstadt, Germany) and analyzed using Sequence Analysis software (Vers. 5.1; ABI) and sequence trace alignment software (SeqMan; DNASTAR, Madison, WI, USA).

### 2.6. Pyrosequencing Assay Design

For AEI verification on an independent sample-set, the pyrosequencing allele quantification method was selected. Scleral tissue was used to analyze the SNPs at the DNA allele level. RNA extracted from retina was used to check allelic imbalance. All primers and assays were designed with PyroMark Assay Design Software 2.0 (QIAGEN, Hilden, Germany) using allele quantification (AQ) settings (Table S2). Primers were checked for off-target pairing with the BLAST-like alignment tool (BLAT) https://genome.ucsc.edu/cgi-bin/hgBlat/) [24]. Three independent PCRs at DNA level and four PCRs from two independent single strand cDNA (complementary DNA) syntheses (Transcriptor High Fidelity cDNA Synthesis Kit; Roche, Mannheim, Germany) were performed (technical replicates). Streptavidin Sepharose™ High Performance beads (GE Healthcare, Uppsala, Sweden) were used for biotinylated strand separation. Pyrosequencing reaction was carried out with PyroMark^®^ Gold Q96 Reagents (QIAGEN, Hilden, Germany) using a PyroMark Q96 Workstation (QIAGEN) and a PyroMark Q96 ID (QIAGEN).

## 3. Results

### 3.1. RNA-sequencing

After collecting post-mortem eyes from 17 human donors not affected by any IRD, two samples with best RNA quality determined by the RIN were subjected to RNA sequencing. These experiments generated 54 M and 78 M reads per sample, respectively, with 61% of the reads mapping to the reference genome (GRCh37/hg19) (Table S1). Visual assessment of AEI candidate SNPs was carried out with IGV (Table S3, Figure S1). Either because of dubious SNP calling or due to the poor complexity of some genomic regions, inconsistent read mapping was observed among our list of IRD genes, leading us to discard SNPs in *OPN1LW/MW* but also in the 3′UTR of the genes *CACNA2D4, CRX*, *EMC1*, *PDE6A*, *RP1L1*, and *TUB* (Figure S2).

### 3.2. Allelic expression imbalanceAnalysis on RNA-sequencing Data

Initially, we conducted the bioinformatic analysis on the two locally generated RNA-seq datasets. The approach described in Section 2.5 gave rise to an average of 93 M reads per sample, with 81% of them mapping to the reference genome (GRCh37/hg19) (Table S1). After applying filters, we obtained 867 and 842 imbalanced SNPs belonging to 676 and 680 genes for the whole retinal transcriptome, respectively (Files S1 and S2).

We repeated the same approach with 50 RNA-seq sample-sets previously published by Pinelli et al. (2016) [19]. In this dataset, we observed 5,856 AEI SNPs in 3,217 genes (File S3). These numbers were filtered down to 4,596 imbalanced SNPs in 702 genes when excluding single AEI hits.

When focusing exclusively on known IRD genes (Retnet, http://www.sph.uth.tmc.edu/RetNet/) [1], we detected 194 SNPs in 67 IRD genes (File S4). Out of these, 20 were fully or partially associated with an autosomal dominant mode of inheritance. Thirty SNPs in these 20 genes displayed AEI in more than 10% of the samples. SNPs lying in mapping-conflictive regions were excluded, as mentioned in Section 3.1 (Table 1), and the final list of AEI in IRD genes is listed in Table 1. We calculated the kurtosis coefficient for every SNP to infer whether the sample ratios found for each SNP were generally well clustered around the average (Table 1). It was found that the larger the kurtosis coefficient (larger than three), the more grouped the samples ratios for a given SNP, suggesting a single molecular cause for the observed imbalance.

Upon this data, we realized that many SNPs, such as rs3130 in *PROM1* (21 samples show allelic ratios higher than 1.5, Table 1) had all or most of their samples’ allelic ratios tilted towards either below 0.66 or above 1.5 thresholds. This extreme biased finding pointed out that this feature occurs when the SNP is in LD with the imbalanced causal variant, or when it belongs to a broader haplotype responsible for AEI. Focusing on autosomal dominant associated IRD genes that we retrieved from HaploReg v4.1 (http://archive.broadinstitute.org/mammals/haploreg/haploreg.php) [25], the regulatory characteristics of the SNPs in LD with the SNPs shown in Table 1 and Table S5.

**Figure 2:**
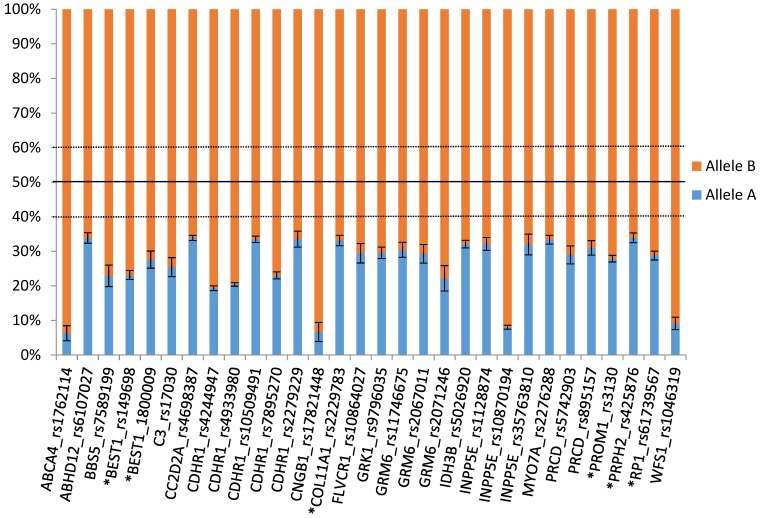
Representation of the relative allelic percentage (mean ± standard error of the mean or SEM). Shown are those SNPs in inherited retinal diseases (IRD) genes displaying allelic expression imbalance (AEI) frequencies higher than 10% in the 52 RNA-seq sample-set. Genes associated with autosomal dominant inherited IRDs are marked with an asterisk. Allelic balance is delimited within the 40–60% range.

Finally, to estimate the strength of the imbalance, we obtained the absolute mean ratio (Table 1) and the relative allelic percentage (Figure 2). For instance, in *BEST1* (rs149698), we found six samples where the ratio was biased towards >1.5 and only one with a ratio <0.66, making a total of seven samples showing AEI out of 16 heterozygous samples for this SNP. In this very example, the absolute mean ratio, calculated upon orientating all samples towards the allele with the lesser read counts (A) divided by the allele with most read counts fraction (B), was equal to 0.31 ± 0.1168 or 23.1–76.9% allelic percentage (Figure 2, Table 1, Table S4). Kurtosis analysis in this very example suggests a diffuse molecular cause with 0.4463 ± 1.587 (Table 1).

### 3.3. Pyrosequencing Confirmation

After obtaining a reliable and comprehensive variant list from RNA-seq AEI analysis (Table 1), we carried on with an independent approach to validate these findings. For this, we used genomic DNA extracted out of scleral tissue and retinal RNA from 17 different healthy eye donors and checked for heterozygosity of the candidate variants. For some of the SNPs, the corresponding pyrosequencing assays were unreliable due to insufficient quality or poor performance of the pyrosequencing probes, yet for the majority of experiments the allele quantification (AQ) method could be used successfully (Table S3). For three of these genes (*CDHR1*, *PROM1*, *BEST1*) we could prove AEI at the RNA level, whereas DNA assessment indicated allele balance either for the candidate SNP and/or analyzed SNPs.

*CDHR1* was the first gene to be analyzed, as it presented the highest AEI frequency according to our RNA-seq data. SNP rs4933980 showed AEI in all heterozygous samples, whereas at the DNA level both alleles remained balanced (Figures S3A and S4). According to the RNA-seq data (Figure 2), the relative allele percentage in AEI samples for rs4933980 lies within the 35.11–64.89% ±0.99 range, whereas in our pyrosequencing analysis the margin was more variable and enlarged to 31.64A–68.36T% ± 0.79.

**Figure 3:**
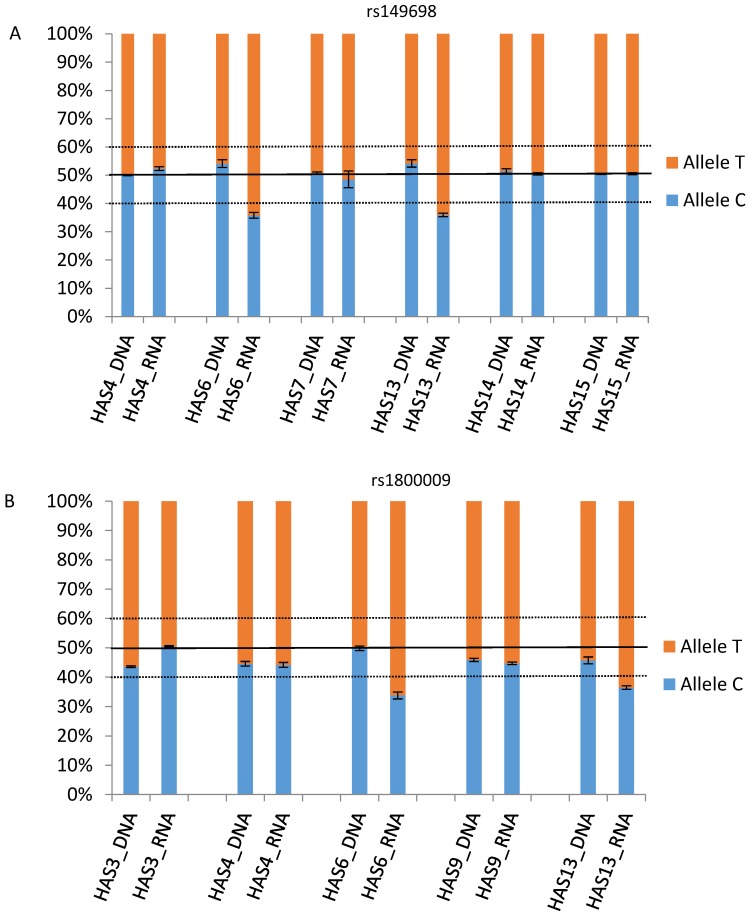
Relative allele percentages for SNPs rs149698 and rs1800009in *BEST1* at the DNA and RNA levels. Allelic balance is delimited within the 40–60% range. Error bars correspond to the standard of the mean (SEM) of allele A. (**A**) *BEST1* rs149698 pyrosequencing results. Allele percentages at the DNA level remained close to 50%. At the RNA level, there was a bias in HAS6 and HAS13 in favor of allele T. (**B**) *BEST1* rs1800009 pyrosequencing results. Similar to rs149698, the DNA levels remained balanced, whereas at the RNA level again HAS6 and HAS13 allele T were overrepresented.

Following the same procedure, we obtained similar results for two autosomal dominant acting genes. In the case of *PROM1*, the pyrosequencing assay for rs3130 showed poor performance. Nonetheless, we analyzed another SNP, rs7686732, for which we found one heterozygous sample to be imbalanced at the RNA level while the remaining were balanced at the DNA level (Figures S3B and S4). Next, we focused on *BEST1*. According to the RNA-seq data we generated, HAS13 displayed AEI throughout the whole transcript (Figure 3, Figure S4). Thus, for this gene we prepared two pyrosequencing assays focusing on rs149698 and rs1800009. In both cases we detected AEI in two samples (HAS6 and HAS13) with balanced allelic ratios at the DNA level. For both SNPs we calculated a mean allele percentage for those samples with AEI obtaining 35.9C–64.1T% and 35.1C–64.9T%, respectively. Yet, we did observed differences in the allelic percentage with respect to the RNA-seq (23.1–76.9% in rs149698 and 27.7–72.3%in rs1800009).

## 4. Discussion

IRDs represent a genetically very heterogeneous group of diseases with all modes of inheritance and a large number of genes involved in the development of a pathological phenotype. However, until recent efforts to better understand retinal transcriptome, little was known about their genomic regulation. In an attempt to improve our comprehension, we investigated the indirect impact of common *cis*-regulatory variants on the whole retinal transcriptome as well as in the context of IRD genes by means of AEI tracking. To do so, we annotated transcribed SNPs that undergo AEI. Out of 50 RNA-seq samples derived from healthy retina donors, we found 5,856 AEI SNPs in 3,217 genes (4,596 imbalanced SNPs in 702 genes when excluding single AEI hits (File S3)) [19]. Focusing the analysis on IRD genes from 52 retinal transcriptomes, we found that 194 SNPs in 67 IRD genes were imbalanced, although as described in Section 3.1, some of them remain dubious. Further filtering indicated that 30 SNPs in 20 IRD genes show AEI with frequencies higher than 10% in the sample population (Figure 2, Table 1).

As a proof of concept, we checked the reliability of our approach by initially confirming that *CDHR1* (Figure S3A), the gene with the highest AEI frequency, indeed shows AEI as confirmed by pyrosequencing. We further extended this approach to other IRD genes associated with autosomal recessive but also with *COL11A1*, *PRPH2*, *PROM1*, *RP1* and *BEST1*, known to underlie an autosomal dominant mode of inheritance. Out of these last five genes, we also validated the results of imbalanced gene expression in *PROM1* and *BEST1* (Figure S3B, Figure 2).

Defects in *COL11A1* are known to cause Stickler and Marshall syndromes. Among other symptoms, these are characterized by radial perivascular retinal degeneration, but to our best knowledge there are no publications specifically reporting variable retinal phenotypes founding such patients and families [26,27]. In contrast, mutations in *PRPH2* are known to cause a variety of retinal phenotypes and have been described in subjects with autosomal dominant adRP, cone dystrophy, macular dystrophy, and digenic retinitis pigmentosa. Interestingly, numerous cases with IP and VE have been reported for this gene [28,29]. Likewise, for *PROM1*, associated with Stargardt disease type 4 and dominant macular dystrophy, IP and VE seems to be a common trait linked to mutations in this gene [30]. *RP1* is one of the most prevalent genes related to adRP. As in other genes, for *RP1* IP and VE phenomena have been reported [31]. Finally, mutations in *BEST1* give rise to autosomal recessive and dominant bestrophinopathies, including best disease, autosomal dominant adult-onset vitelliform macular dystrophy, and autosomal dominant vitreoretinochoroidopathy. For this gene, the literature also describes various examples with IP or VE [32,33,34].

As shown in Table 1 and Figure 2, *PRPH2* presents with a strong AEI (9.1–98.9% ± 0.0357) with a quite diverse reason for AEI (kurtosis−1.368, SEK 1.587). In the case of *PROM1*, the relative allele percentage is 25.6–74.4% ± 0.017; nonetheless its kurtosis (4.62, SEK 0.9719) indicates a unique source of AEI. The analysis in *RP1* resulted in a 28.8–71.2% ± 0.0256 imbalance percentage with high kurtosis (10.86, SEK 0.9178), pointing to a single AEI molecular cause. For *BEST1* (rs149698), we detected an allelic percentage of 23.1–76.9% ± 0.044 by means of RNA-seq, levels slightly more extreme than when confirmed by pyrosequencing (35.9C–64.1T%); however, it seems that there should be several underlying variants for the observed imbalance (kurtosis 0.4463, SEK1.587).

We found out that the SNPs belonging to the last four genes (among others) are prone to bias their ratio towards one direction (Table 1). This suggests that the variant or haplotype responsible for AEI is in LD with the listed SNP. Within this study, we collected all variants and their known regulatory features in LD (r^2^ > 0.8) with rs425876 (*PRPH2*), rs3130 (*PROM1*), rs61739567 (*RP1*),and rs149698 (*BEST1*) from HaploReg v4.1(Table S5) [25].

To verify whether or not these or other SNPs participate in any *cis*-regulatory mechanism in these genes, and to determine the overall gain or loss of transcript quantity, further functional research is needed. Crossing these results with genotypes of clinical cases may help to analyse and explain the observed variable phenotype and may in turn be used as prognostic marker.

## 5. Conclusions

Phenotypical variability is a well-reported hallmark in IRDs. In this work, we analyzed 52 healthy retinal transcriptomes to elucidate which genes could contribute to such variability in terms of AEI. We identified 194 SNPs in 67 IRD genes, out of which 30 SNPs belonging to 20 IRD genes display AEI in a quite common fashion, with at least 10% of AEI frequency. Interestingly, *PRPH2*, *PROM1*, *RP1*, and *BEST1*, genes associated with autosomal dominant forms of IRD that are present in this selective list, are known to be affected by IP and VE. To prove the relevance of AEI in clinical cases linked to these genes, further investigation concerning the underlying molecular cause for such frequent phenomena and their segregation pattern within affected families is still needed. With this comprehensive list of AEI SNPs, we hope to contribute to the elucidation and understanding of the mechanisms underlying these phenomena in IRD, and to induce reassessment of clinical cases by ophthalmic clinicians and researchers in the future.

## Figures and Tables

**Table 1:** SNPs in inherited retinal diseases (IRD) genes displaying allelic expression imbalance (AEI) frequencies higher than 10% in the 52 RNA-seq dataset. Highlighted with an asterisk are those genes known to be associated with autosomal dominant IRD. Kurtosis was estimated to stress those SNPs where imbalance was consistent with a single molecular cause (kurtosis ≥3 indicates closer distribution of the data points towards the mean; standard error of kurtosis is provided as SEK). Standard error of the mean (SEM) of the allelic ratio was also calculated.

Gene Symbol_ SNP	Samples in Top Mean ±2*SD Ratios	Heterozygous Samples	Allelic Ratio <0.66	Allelic Ratio >1.5	Sum Imbalanced Samples	AEI Frequency in Heterozygous Samples	AEI Frequency in 52 Samples	Absolute Mean Allelic Ratio	SEM Allelic Ratio	Kurtosis	SEK
*ABCA4*_rs1762114	7	16	0	8	8	50.0%	15.4%	0.090	0.069	7.636	1.481
*ABHD12*_rs6107027	1	20	11	1	12	60.0%	23.1%	0.536	0.050	8.709	1.232
*BBS5*_rs7589199	3	11	5	3	8	72.7%	15.4%	0.354	0.101	−2.19	1.481
*BEST1_rs149698	4	22	1	6	7	31.8%	13.5%	0.310	0.044	0.4463	1.587
*BEST1_1800009	2	20	4	2	6	30.0%	11.5%	0.412	0.091	−1.865	1.741
*C3*_rs17030	2	29	2	6	8	27.6%	15.4%	0.385	0.087	−0.5529	1.481
*CC2D2A*_rs4698387	1	17	11	0	11	64.7%	21.2%	0.519	0.032	0.3474	1.279
*CDHR1*_rs4244947	16	23	21	2	23	100.0%	44.2%	0.247	0.020	0.9735	0.9348
*CDHR1*_rs4933980	17	21	21	0	21	100.0%	40.4%	0.260	0.016	0.3758	0.9719
*CDHR1*_rs10509491	1	20	11	0	11	55.0%	21.2%	0.513	0.039	−0.1936	1.279
*CDHR1*_rs7895270	6	16	10	0	10	62.5%	19.2%	0.308	0.038	1.261	1.334
*CDHR1*_rs2279229	1	15	7	1	8	53.3%	15.4%	0.541	0.076	7.562	1.481
*CNGB1*_rs17821448	10	30	1	10	11	36.7%	21.2%	0.158	0.147	11	1.279
*COL11A1_rs2229783	2	19	2	7	9	47.4%	17.3%	0.516	0.058	1.01	1.4
*FLVCR1*_rs10864027	1	24	5	1	6	25.0%	11.5%	0.454	0.095	1.819	1.741
*GRK1*_rs9796035	3	21	10	2	12	57.1%	23.1%	0.449	0.058	0.5881	1.232
*GRM6*_rs11746675	2	32	5	4	9	28.1%	17.3%	0.474	0.074	1.466	1.4
*GRM6*_rs2067011	1	29	4	3	7	24.1%	13.5%	0.455	0.091	0.6681	1.587
*GRM6*_rs2071246	3	22	2	4	6	27.3%	11.5%	0.344	0.125	−3.026	1.741
*IDH3B*_rs5026920	1	12	8	0	8	66.7%	15.4%	0.482	0.045	0.2172	1.481
*INPP5E*_rs1128874	2	27	8	1	9	33.3%	17.3%	0.500	0.062	5.798	1.154
*INPP5E*_rs10870194	2	24	0	24	24	100.0%	46.2%	0.092	0.016	0.3604	1.4
*INPP5E*_rs35763810	3	20	0	6	6	30.0%	11.5%	0.513	0.099	6.436	1.587
*MYO7A*_rs2276288	3	17	13	1	14	82.4%	26.9%	0.522	0.044	2.193	1.014
*PRCD*_rs5742903	1	25	7	2	9	36.0%	17.3%	0.457	0.085	0.9821	1.481
*PRCD*_rs895157	1	17	6	1	7	41.2%	13.5%	0.474	0.069	1.585	1.481
*PROM1_rs3130	8	20	0	19	19	95.0%	36.5%	0.401	0.032	−1.368	1.587
*PRPH2_rs425876	6	14	1	7	8	57.1%	15.4%	0.529	0.057	5.791	1.4
*RP1_rs61739567	2	11	0	8	8	72.7%	15.4%	0.438	0.049	10.86	0.9178
*WFS1*_rs1046319	1	12	0	7	7	58.3%	13.5%	0.111	0.046	5.809	1.741

## Supplementary Materials

The following material is available online at www.mdpi.com/2073-4425/8/10/283/s1, Figure S1: IGV visual inspection on RNA-seq AEI SNP allelic imbalance, Figure S2: IGV visual inspection on candidate SNP regions, Figure S3: Relative allele percentages for SNPs rs4933980 in CDHR1 andrs7686732, Figure S4: Pyrograms, Table S1: Biological information of collected eye donor samples, Table S2: List of pyrosequencing and PCR-Sanger primer, Table S3: RNA-seq AEI SNP list, Table S4: Relative allelic percentage in IRD genes, Table S5: List of SNPs in LD with AEI candidate SNPs. File S1: HAS4 raw AEI RNA-seq, File S2: HAS13 raw AEI RNA-seq, File S3: AEI in 50 samples, File S4: IRD SNPs in 52 samples.
