# Supplementary material for: Allelic Expression Imbalance in the Human Retinal Transcriptome and Potential Impact on Inherited Retinal Diseases
Source: Genes (Basel). 2017 Oct 20;8(10):283. doi: 10.3390/genes8100283 (PMC5664133; doi:10.3390/genes8100283)
Supplement: Supplementary file 1 [file genes-08-00283-s001.zip › Figure S1.IGV visual inspection of RNA-seq samples with respect to allelic expression imbalanced (AEI) SNP.docx]

1. *BEST1*: rs149698


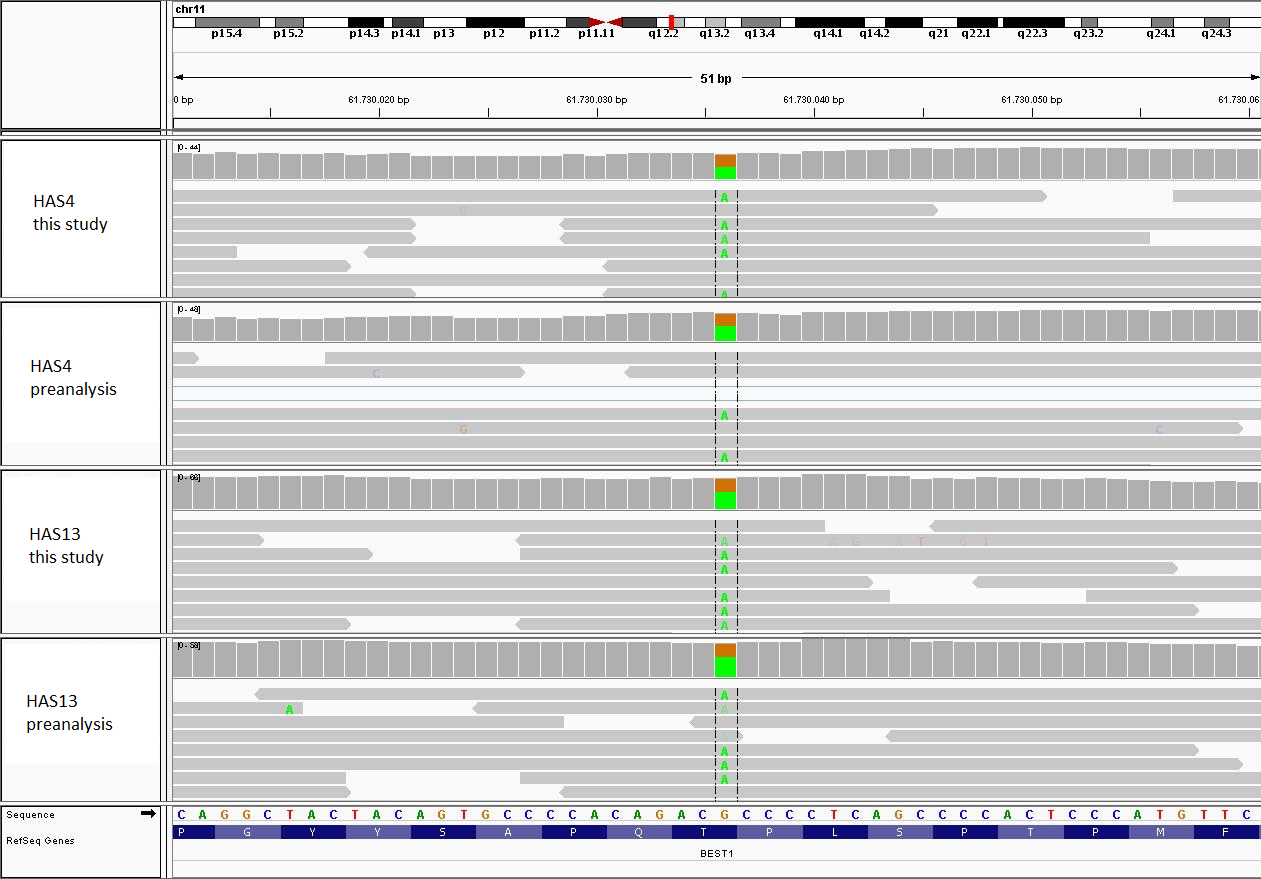


A: 56 %

G: 44 %

A: 60 %

G: 40 %

A: 56 %

G: 44 %

A: 50 %

G: 50 %

1. *BEST1*: rs1800009


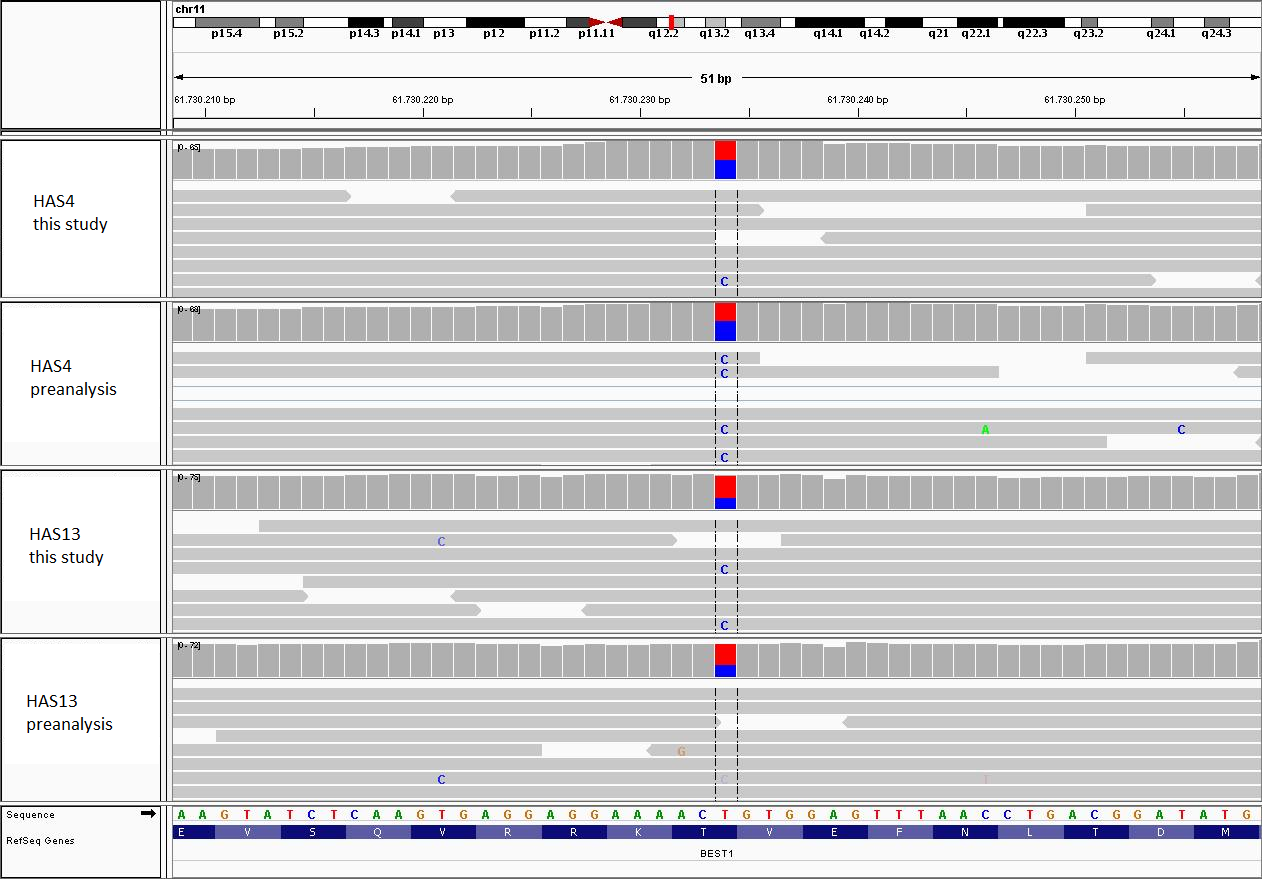


C: 33 %

G: 67 %

C: 37 %

G: 63 %

C: 53 %

G: 47 %

C: 51 %

G: 49 %

1. *GRK1*: rs9796035


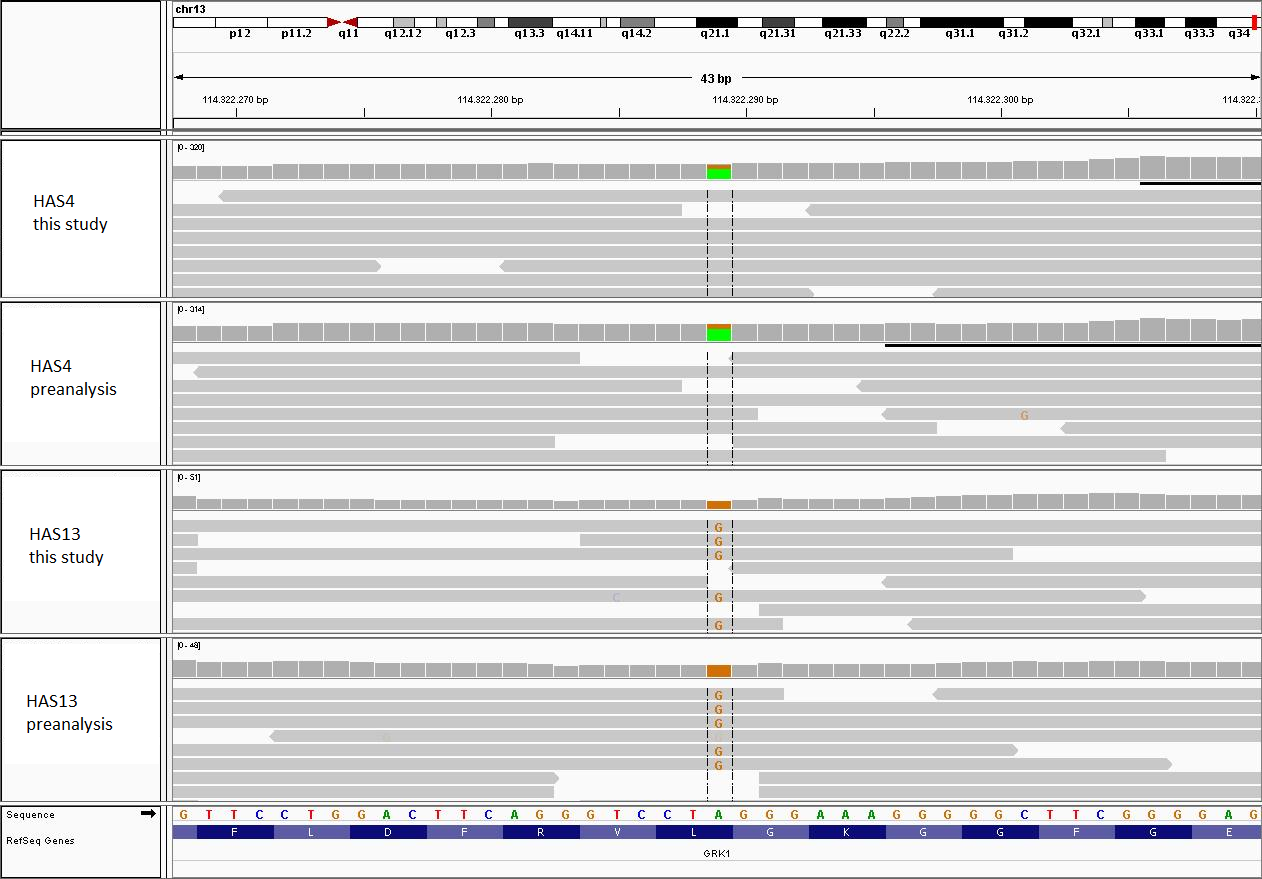


A: 71 %

G: 29 %

A: 70 %

G: 30 %

1. *INNP5E*: rs10870194


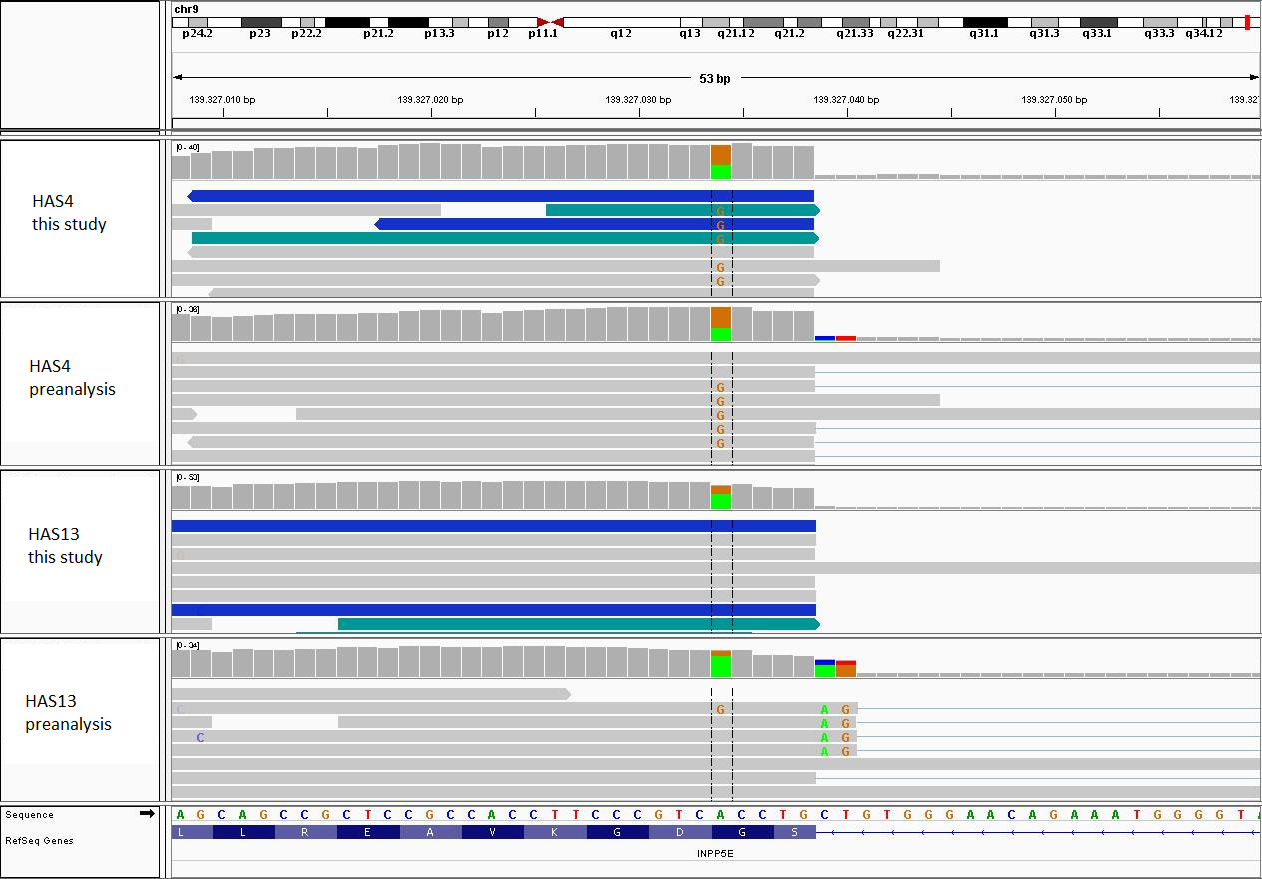


A: 39 %

G: 61 %

A: 41 %

G: 59 %

A: 78 %

G: 22 %

A: 66 %

G: 34 %

1. *INPP5E*: rs33982662


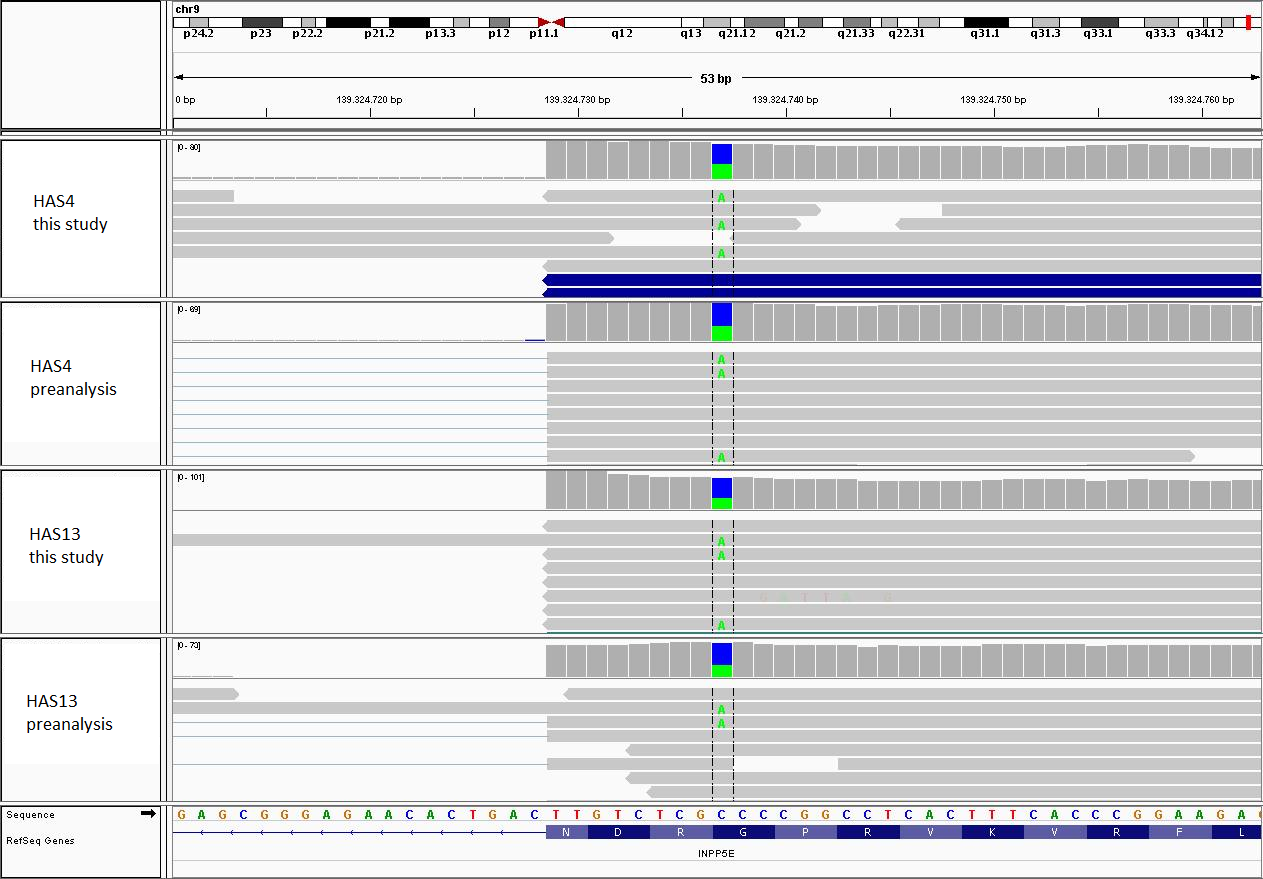


A: 40 %

C: 60 %

A: 43 %

C: 57 %

A: 35 %

C: 65 %

A: 35 %

C: 65 %

1. *PROM1*: rs3130


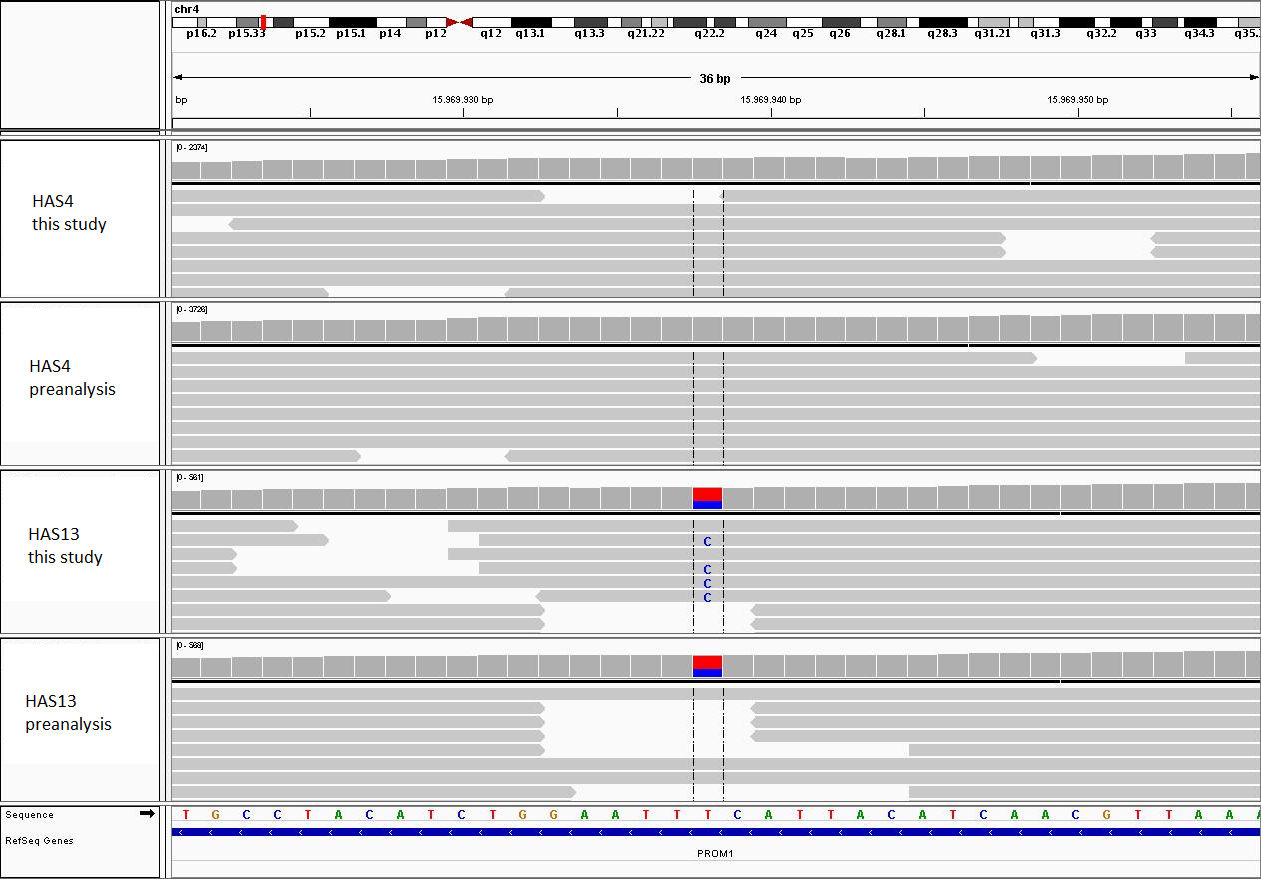


A: 39 %

C: 61 %

A: 38 %

C: 62 %

**Figure S1.** Integrative Genomics Viewer (IGV) visual inspection of RNA-seq samples with respect to allelic expression imbalanced (AEI) SNP (allelic proportion is shown in different colors attending to each nucleotide: T (red), C (blue), A (green), G (brown). Indicated are the relative allele percentages for each SNP and IGV snapshots of those genes with AEI SNPs in the three RNA-seq samples. The two first rows correspond to eye sample HAS4 (first row: processing according to section 2.4, second row: Microarray Facility Tuebingen Services processing). The next two rows correspond to eye donor sample HAS13 (first row: processing according to section 2.4, second row: MTF services processing). Represented are: A) *BEST1* rs149698; B) *BEST1* rs1800009; C) *GRK1* rs9796035; D) *INNP5* rs10870194; E) *INPP5E* rs33982662; F) *PROM1* rs3130.
