# Supplementary material for: Allelic Expression Imbalance in the Human Retinal Transcriptome and Potential Impact on Inherited Retinal Diseases
Source: Genes (Basel). 2017 Oct 20;8(10):283. doi: 10.3390/genes8100283 (PMC5664133; doi:10.3390/genes8100283)
Supplement: Supplementary file 1 [file genes-08-00283-s001.zip › Figure S2. IGV visual inspection on candidate SNP regions.docx]

*EMC1:* MAF does not fit with our SNP frequency, besides new variant are created

*
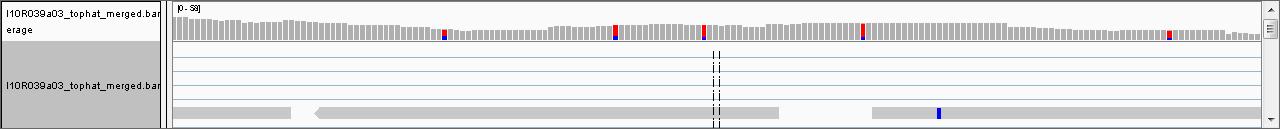
*

*CACNA2D4*: highly repetitive region at 3’UTR


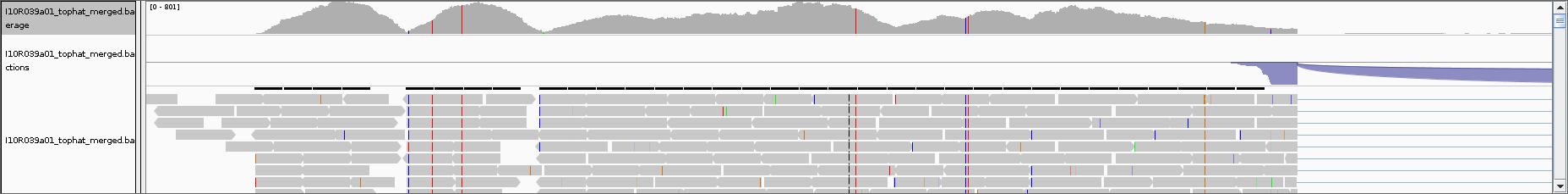


*CRX:* inconsistent mapping of reads leads to false splicing events

*
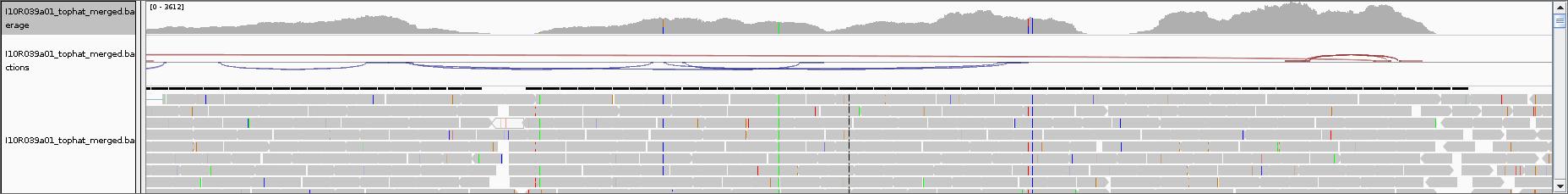
*

*PDE6A:* inconsistent mapping of reads leads to false splicing events

*
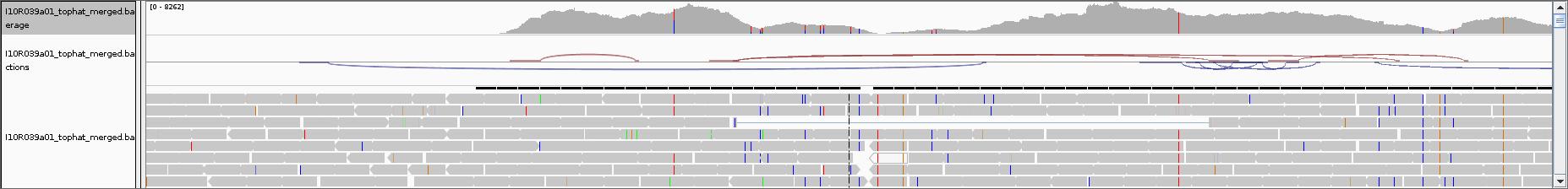
*

*OPN1LW/MW*: genes sharing high sequence identity results in misalignment, i.e. *OPN1LW* reads are mapped to *OPN1MW* and vice versa

*
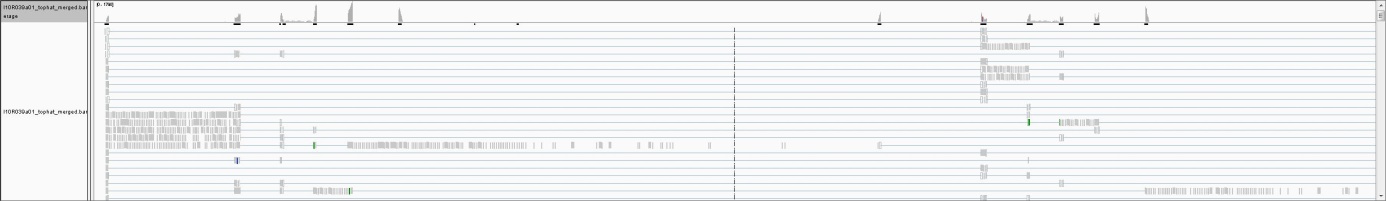
*

*RP1L1:* inconsistent mapping of reads leads to false splicing events

*
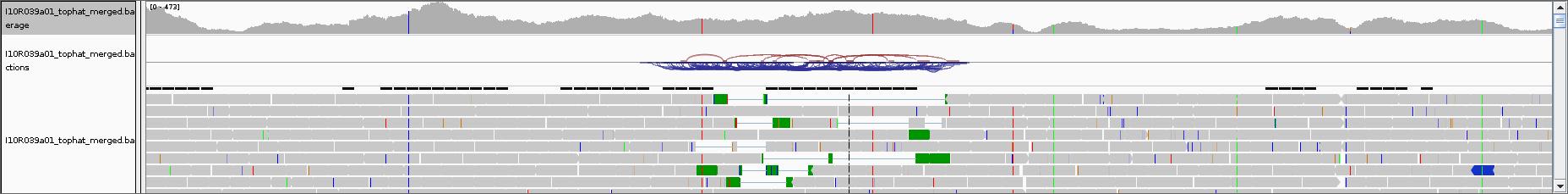
*

*TUB:* SNP positioned in T-rich region (17 T in a row)

*
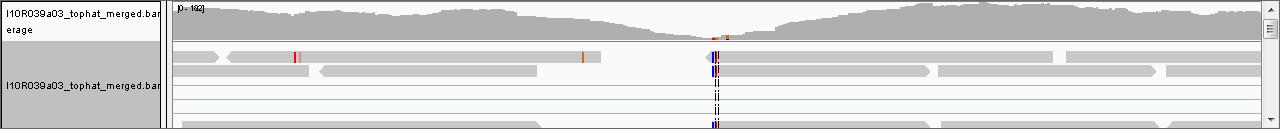
*

**Figure S2.** Integrative Genomics Viewer (IGV) visual inspection on candidate SNP regions.
