# Supplementary material for: Allelic Expression Imbalance in the Human Retinal Transcriptome and Potential Impact on Inherited Retinal Diseases
Source: Genes (Basel). 2017 Oct 20;8(10):283. doi: 10.3390/genes8100283 (PMC5664133; doi:10.3390/genes8100283)
Supplement: Supplementary file 1 [file genes-08-00283-s001.zip › Figure S3. Pyrosequencing relative allele percentages in CDHR1 and PROM1.docx]

*PROM1*

*CDHR1*

B

A

**Figure S3.** Relative allele percentages for SNPs rs4933980 in CDHR1 and rs7686732 in PROM1 on DNA and RNA level. Error bars correspond to standerd error of the mean (SEM) of allele. **A)** CDHR1 rs4933980 pyrosequencing results. Whereas allele percentage at DNA level remained close to 50%, a clear bias was observed in favor of allele T on RNA level. **B)** PROM1 rs7686732 pyrosequencing results. Only HAS4 was found to be heterozygous for this SNP. DNA alleles percentage stayed below the 40-60% threshold, whereas at RNA level allele C corresponded to 60.25% and allele G to 39.75%.
