# Supplementary material for: Allelic Expression Imbalance in the Human Retinal Transcriptome and Potential Impact on Inherited Retinal Diseases
Source: Genes (Basel). 2017 Oct 20;8(10):283. doi: 10.3390/genes8100283 (PMC5664133; doi:10.3390/genes8100283)
Supplement: Supplementary file 1 [file genes-08-00283-s001.zip › Figure S4. Pyrograms.docx]

*BEST1*: rs149698

BEST1_Ex9_18.06.15 - Well B11

Entry: BEST1_Ex9_rs149698

Sample: 2R10

Position1: C: 100.0% / T: 0.0% (Passed)

BEST1_Ex9_18.06.15 - Well B12

Entry: BEST1_Ex9_rs149698

Sample: 2R-

Position1: C: 0.0% / T: 100.0% (Failed)

BEST1_Ex9_18.06.15 - Well C2

Entry: BEST1_Ex9_rs149698

Sample: D3

Position1: C: 100.0% / T: 0.0% (Passed)

BEST1_Ex9_18.06.15 - Well C3

Entry: BEST1_Ex9_rs149698

Sample: D11

Position1: C: 0.0% / T: 100.0% (Passed)

BEST1_Ex9_18.06.15 - Well C4

Entry: BEST1_Ex9_rs149698

Sample: D--

Position1: C: 0.0% / T: 100.0% (Failed)

BEST1_Ex9_18.06.15 - Well C6

Entry: BEST1_Ex9_rs149698

Sample: 1R3

Position1: C: 100.0% / T: 0.0% (Check)

BEST1_Ex9_18.06.15 - Well C7

Entry: BEST1_Ex9_rs149698

Sample: 1R11

Position1: C: 0.0% / T: 100.0% (Passed)

BEST1_Ex9_18.06.15 - Well C8

Entry: BEST1_Ex9_rs149698

Sample: 1R--

Position1: C: 0.0% / T: 100.0% (Failed)

BEST1_Ex9_18.06.15 - Well C10

Entry: BEST1_Ex9_rs149698

Sample: 2R3

Position1: C: 100.0% / T: 0.0% (Passed)

BEST1_Ex9_18.06.15 - Well C11

Entry: BEST1_Ex9_rs149698

Sample: 2R11

Position1: C: 0.0% / T: 100.0% (Passed)

BEST1_Ex9_18.06.15 - Well C12

Entry: BEST1_Ex9_rs149698

Sample: 2R--

Position1: C: 0.0% / T: 100.0% (Failed)

BEST1_Ex9_18.06.15 - Well D2

Entry: BEST1_Ex9_rs149698

Sample: D4

Position1: C: 50.0% / T: 50.0% (Passed)

BEST1_Ex9_18.06.15 - Well D3

Entry: BEST1_Ex9_rs149698

Sample: D12

Position1: C: 100.0% / T: 0.0% (Passed)

BEST1_Ex9_18.06.15 - Well D6

Entry: BEST1_Ex9_rs149698

Sample: 1R4

Position1: C: 52.9% / T: 47.1% (Passed)

BEST1_Ex9_18.06.15 - Well D7

Entry: BEST1_Ex9_rs149698

Sample: 1R12

Position1: C: 100.0% / T: 0.0% (Passed)

BEST1_Ex9_18.06.15 - Well D10

Entry: BEST1_Ex9_rs149698

Sample: 2R4

Position1: C: 49.7% / T: 50.3% (Passed)

BEST1_Ex9_18.06.15 - Well D11

Entry: BEST1_Ex9_rs149698

Sample: 2R12

Position1: C: 100.0% / T: 0.0% (Passed)

BEST1_Ex9_18.06.15 - Well E2

Entry: BEST1_Ex9_rs149698

Sample: D5

Position1: C: 100.0% / T: 0.0% (Passed)

BEST1_Ex9_18.06.15 - Well E3

Entry: BEST1_Ex9_rs149698

Sample: D13

Position1: C: 49.6% / T: 50.4% (Passed)

BEST1_Ex9_18.06.15 - Well E6

Entry: BEST1_Ex9_rs149698

Sample: 1R5

Position1: C: 100.0% / T: 0.0% (Passed)

BEST1_Ex9_18.06.15 - Well E7

Entry: BEST1_Ex9_rs149698

Sample: 1R13

Position1: C: 31.8% / T: 68.2% (Passed)

BEST1_Ex9_18.06.15 - Well E10

Entry: BEST1_Ex9_rs149698

Sample: 2R5

Position1: C: 100.0% / T: 0.0% (Passed)

BEST1_Ex9_18.06.15 - Well E11

Entry: BEST1_Ex9_rs149698

Sample: 2R13

Position1: C: 32.8% / T: 67.2% (Passed)

BEST1_Ex9_18.06.15 - Well F2

Entry: BEST1_Ex9_rs149698

Sample: D6

Position1: C: 49.4% / T: 50.6% (Passed)

BEST1_Ex9_18.06.15 - Well F3

Entry: BEST1_Ex9_rs149698

Sample: D14

Position1: C: 49.6% / T: 50.4% (Passed)

BEST1_Ex9_18.06.15 - Well F6

Entry: BEST1_Ex9_rs149698

Sample: 1R6

Position1: C: 29.7% / T: 70.3% (Passed)

BEST1_Ex9_18.06.15 - Well F7

Entry: BEST1_Ex9_rs149698

Sample: 1R14

Position1: C: 48.5% / T: 51.5% (Passed)

BEST1_Ex9_18.06.15 - Well F10

Entry: BEST1_Ex9_rs149698

Sample: 2R6

Position1: C: 32.7% / T: 67.3% (Passed)

BEST1_Ex9_18.06.15 - Well F11

Entry: BEST1_Ex9_rs149698

Sample: 2R14

Position1: C: 50.9% / T: 49.1% (Passed)

BEST1_Ex9_18.06.15 - Well G2

Entry: BEST1_Ex9_rs149698

Sample: D7

Position1: C: 52.2% / T: 47.8% (Passed)

BEST1_Ex9_18.06.15 - Well G3

Entry: BEST1_Ex9_rs149698

Sample: D15

Position1: C: 52.8% / T: 47.2% (Passed)

BEST1_Ex9_18.06.15 - Well G6

Entry: BEST1_Ex9_rs149698

Sample: 1R7

Position1: C: 46.6% / T: 53.4% (Passed)

BEST1_Ex9_18.06.15 - Well G7

Entry: BEST1_Ex9_rs149698

Sample: 1R15

Position1: C: 50.4% / T: 49.6% (Passed)

BEST1_Ex9_18.06.15 - Well G10

Entry: BEST1_Ex9_rs149698

Sample: 2R7

Position1: C: 52.9% / T: 47.1% (Passed)

BEST1_Ex9_18.06.15 - Well G11

Entry: BEST1_Ex9_rs149698

Sample: 2R15

Position1: C: 49.4% / T: 50.6% (Passed)

BEST1_Ex9_18.06.15 - Well H2

Entry: BEST1_Ex9_rs149698

Sample: D8

Position1: C: 100.0% / T: 0.0% (Passed)

BEST1_Ex9_18.06.15 - Well H3

Entry: BEST1_Ex9_rs149698

Sample: D16

Position1: C: 0.0% / T: 100.0% (Passed)

BEST1_Ex9_18.06.15 - Well H6

Entry: BEST1_Ex9_rs149698

Sample: 1R8

Position1: C: 100.0% / T: 0.0% (Passed)

BEST1_Ex9_18.06.15 - Well H7

Entry: BEST1_Ex9_rs149698

Sample: 1R16

Position1: C: 0.0% / T: 100.0% (Passed)

BEST1_Ex9_18.06.15 - Well H10

Entry: BEST1_Ex9_rs149698

Sample: 2R8

Position1: C: 100.0% / T: 0.0% (Passed)

BEST1_Ex9_18.06.15 - Well H11

Entry: BEST1_Ex9_rs149698

Sample: 2R16

Position1: C: 0.0% / T: 100.0% (Passed)

4.6.15_BEST1 RNA - Well A1

Entry: BEST1_Ex9_rs149698

Sample: 1R4

Position1: C: 53.3% / T: 46.7% (Passed)

4.6.15_BEST1 RNA - Well A3

Entry: BEST1_Ex9_rs149698

Sample: 2R4

Position1: C: 58.8% / T: 41.2% (Passed)

4.6.15_BEST1 RNA - Well A5

Entry: BEST1_Ex9_rs149698

Sample: 3R4

Position1: C: 0.0% / T: 100.0% (Failed)

4.6.15_BEST1 RNA - Well A7

Entry: BEST1_Ex9_rs149698

Sample: D1

Position1: C: 0.0% / T: 100.0% (Failed)

4.6.15_BEST1 RNA - Well B1

Entry: BEST1_Ex9_rs149698

Sample: 1R6

Position1: C: 33.5% / T: 66.5% (Passed)

4.6.15_BEST1 RNA - Well B3

Entry: BEST1_Ex9_rs149698

Sample: 2R6

Position1: C: 44.5% / T: 55.5% (Passed)

4.6.15_BEST1 RNA - Well B5

Entry: BEST1_Ex9_rs149698

Sample: 3R6

Position1: C: 29.7% / T: 70.3% (Passed)

4.6.15_BEST1 RNA - Well B7

Entry: BEST1_Ex9_rs149698

Sample: D4

Position1: C: 0.0% / T: 100.0% (Failed)

4.6.15_BEST1 RNA - Well C1

Entry: BEST1_Ex9_rs149698

Sample: 1R7

Position1: C: 46.8% / T: 53.2% (Passed)

4.6.15_BEST1 RNA - Well C3

Entry: BEST1_Ex9_rs149698

Sample: 2R7

Position1: C: 50.4% / T: 49.6% (Passed)

4.6.15_BEST1 RNA - Well C5

Entry: BEST1_Ex9_rs149698

Sample: 3R7

Position1: C: 44.5% / T: 55.5% (Passed)

4.6.15_BEST1 RNA - Well C7

Entry: BEST1_Ex9_rs149698

Sample: D6

Position1: C: 0.0% / T: 100.0% (Failed)

4.6.15_BEST1 RNA - Well D1

Entry: BEST1_Ex9_rs149698

Sample: 1R13

Position1: C: 70.2% / T: 29.8% (Passed)

4.6.15_BEST1 RNA - Well D3

Entry: BEST1_Ex9_rs149698

Sample: 2R13

Position1: C: 38.7% / T: 61.3% (Passed)

4.6.15_BEST1 RNA - Well D5

Entry: BEST1_Ex9_rs149698

Sample: 3R13

Position1: C: 33.5% / T: 66.5% (Passed)

4.6.15_BEST1 RNA - Well D7

Entry: BEST1_Ex9_rs149698

Sample: D7

Position1: C: 0.0% / T: 100.0% (Failed)

4.6.15_BEST1 RNA - Well E1

Entry: BEST1_Ex9_rs149698

Sample: 1R14

Position1: C: 61.2% / T: 38.8% (Passed)

4.6.15_BEST1 RNA - Well E3

Entry: BEST1_Ex9_rs149698

Sample: 2R14

Position1: C: 49.2% / T: 50.8% (Passed)

4.6.15_BEST1 RNA - Well E5

Entry: BEST1_Ex9_rs149698

Sample: 3R14

Position1: C: 50.0% / T: 50.0% (Passed)

4.6.15_BEST1 RNA - Well E7

Entry: BEST1_Ex9_rs149698

Sample: D13

Position1: C: 0.0% / T: 100.0% (Failed)

4.6.15_BEST1 RNA - Well F1

Entry: BEST1_Ex9_rs149698

Sample: 1R15

Position1: C: 49.4% / T: 50.6% (Passed)

4.6.15_BEST1 RNA - Well F3

Entry: BEST1_Ex9_rs149698

Sample: 2R15

Position1: C: 49.7% / T: 50.3% (Passed)

4.6.15_BEST1 RNA - Well F5

Entry: BEST1_Ex9_rs149698

Sample: 3R15

Position1: C: 31.2% / T: 68.8% (Passed)

4.6.15_BEST1 RNA - Well F7

Entry: BEST1_Ex9_rs149698

Sample: D14

Position1: C: 0.0% / T: 100.0% (Failed)

4.6.15_BEST1 RNA - Well G1

Entry: BEST1_Ex9_rs149698

Sample: 1R-

Position1: C: 0.0% / T: 100.0% (Failed)

4.6.15_BEST1 RNA - Well H1

Entry: BEST1_Ex9_rs149698

Sample: 1R--

Position1: C: 100.0% / T: 0.0% (Failed)

4.6.15_BEST1 RNA - Well H3

Entry: BEST1_Ex9_rs149698

Sample: 2R--

Position1: C: 0.0% / T: 100.0% (Failed)

4.6.15_BEST1 RNA - Well H5

Entry: BEST1_Ex9_rs149698

Sample: 3R--

Position1: C: 0.0% / T: 100.0% (Failed)

*BEST1:* rs1800009

BEST1_rs1800009 - Well A1

Entry: BEST1_Ex9_rs1800009

Sample: D1

Position1: C: 0.0% / T: 100.0% (Passed)

BEST1_rs1800009 - Well A2

Entry: BEST1_Ex9_rs1800009

Sample: D9

Position1: C: 46.9% / T: 53.1% (Passed)

BEST1_rs1800009 - Well A3

Entry: BEST1_Ex9_rs1800009

Sample: D17

Position1: C: 4.2% / T: 95.8% (Passed)

BEST1_rs1800009 - Well A5

Entry: BEST1_Ex9_rs1800009

Sample: 1R1

Position1: C: 8.7% / T: 91.3% (Failed)

BEST1_rs1800009 - Well A6

Entry: BEST1_Ex9_rs1800009

Sample: 1R9

Position1: C: 47.1% / T: 52.9% (Passed)

BEST1_rs1800009 - Well A7

Entry: BEST1_Ex9_rs1800009

Sample: 1R17

Position1: C: 4.8% / T: 95.2% (Passed)

BEST1_rs1800009 - Well A9

Entry: BEST1_Ex9_rs1800009

Sample: 2R1

Position1: C: 1.8% / T: 98.2% (Failed)

BEST1_rs1800009 - Well A10

Entry: BEST1_Ex9_rs1800009

Sample: 2R9

Position1: C: 43.4% / T: 56.6% (Passed)

BEST1_rs1800009 - Well A11

Entry: BEST1_Ex9_rs1800009

Sample: 2R17

Position1: C: 5.8% / T: 94.2% (Passed)

BEST1_rs1800009 - Well B1

Entry: BEST1_Ex9_rs1800009

Sample: D2

Position1: C: 0.0% / T: 100.0% (Passed)

BEST1_rs1800009 - Well B2

Entry: BEST1_Ex9_rs1800009

Sample: D10

Position1: C: 5.9% / T: 94.1% (Passed)

BEST1_rs1800009 - Well B3

Entry: BEST1_Ex9_rs1800009

Sample: D-

Position1: C: 0.0% / T: 100.0% (Failed)

BEST1_rs1800009 - Well B5

Entry: BEST1_Ex9_rs1800009

Sample: 1R2

Position1: C: 2.9% / T: 97.1% (Passed)

BEST1_rs1800009 - Well B6

Entry: BEST1_Ex9_rs1800009

Sample: 1R10

Position1: C: 0.0% / T: 100.0% (Failed)

BEST1_rs1800009 - Well B7

Entry: BEST1_Ex9_rs1800009

Sample: 1R--

Position1: C: 0.0% / T: 100.0% (Failed)

BEST1_rs1800009 - Well B9

Entry: BEST1_Ex9_rs1800009

Sample: 2R2

Position1: C: 2.3% / T: 97.7% (Passed)

BEST1_rs1800009 - Well B10

Entry: BEST1_Ex9_rs1800009

Sample: 2R10

Position1: C: 0.0% / T: 100.0% (Failed)

BEST1_rs1800009 - Well B11

Entry: BEST1_Ex9_rs1800009

Sample: 2R--

Position1: C: 0.0% / T: 100.0% (Failed)

BEST1_rs1800009 - Well C1

Entry: BEST1_Ex9_rs1800009

Sample: D3

Position1: C: 42.5% / T: 57.5% (Passed)

BEST1_rs1800009 - Well C2

Entry: BEST1_Ex9_rs1800009

Sample: D11

Position1: C: 3.1% / T: 96.9% (Passed)

BEST1_rs1800009 - Well C5

Entry: BEST1_Ex9_rs1800009

Sample: 1R3

Position1: C: 50.9% / T: 49.1% (Passed)

BEST1_rs1800009 - Well C6

Entry: BEST1_Ex9_rs1800009

Sample: 1R11

Position1: C: 3.3% / T: 96.7% (Passed)

BEST1_rs1800009 - Well C7

Entry: BEST1_Ex9_rs1800009

Sample: 1R-

Position1: C: 0.0% / T: 100.0% (Failed)

BEST1_rs1800009 - Well C9

Entry: BEST1_Ex9_rs1800009

Sample: 2R3

Position1: C: 50.1% / T: 49.9% (Passed)

BEST1_rs1800009 - Well C10

Entry: BEST1_Ex9_rs1800009

Sample: 2R11

Position1: C: 0.7% / T: 99.3% (Passed)

BEST1_rs1800009 - Well C11

Entry: BEST1_Ex9_rs1800009

Sample: 2R-

Position1: C: 0.0% / T: 100.0% (Failed)

BEST1_rs1800009 - Well D1

Entry: BEST1_Ex9_rs1800009

Sample: D4

Position1: C: 41.9% / T: 58.1% (Passed)

BEST1_rs1800009 - Well D2

Entry: BEST1_Ex9_rs1800009

Sample: D12

Position1: C: 7.4% / T: 92.6% (Passed)

BEST1_rs1800009 - Well D5

Entry: BEST1_Ex9_rs1800009

Sample: 1R4

Position1: C: 44.5% / T: 55.5% (Passed)

BEST1_rs1800009 - Well D6

Entry: BEST1_Ex9_rs1800009

Sample: 1R12

Position1: C: 4.1% / T: 95.9% (Passed)

BEST1_rs1800009 - Well D9

Entry: BEST1_Ex9_rs1800009

Sample: 2R4

Position1: C: 48.6% / T: 51.4% (Passed)

BEST1_rs1800009 - Well D10

Entry: BEST1_Ex9_rs1800009

Sample: 2R12

Position1: C: 2.4% / T: 97.6% (Passed)

BEST1_rs1800009 - Well E1

Entry: BEST1_Ex9_rs1800009

Sample: D5

Position1: C: 1.7% / T: 98.3% (Passed)

BEST1_rs1800009 - Well E2

Entry: BEST1_Ex9_rs1800009

Sample: D13

Position1: C: 49.8% / T: 50.2% (Passed)

BEST1_rs1800009 - Well E5

Entry: BEST1_Ex9_rs1800009

Sample: 1R5

Position1: C: 6.6% / T: 93.4% (Passed)

BEST1_rs1800009 - Well E6

Entry: BEST1_Ex9_rs1800009

Sample: 1R13

Position1: C: 37.5% / T: 62.5% (Passed)

BEST1_rs1800009 - Well E9

Entry: BEST1_Ex9_rs1800009

Sample: 2R5

Position1: C: 3.0% / T: 97.0% (Passed)

BEST1_rs1800009 - Well E10

Entry: BEST1_Ex9_rs1800009

Sample: 2R13

Position1: C: 30.8% / T: 69.2% (Passed)

BEST1_rs1800009 - Well F1

Entry: BEST1_Ex9_rs1800009

Sample: D6

Position1: C: 52.7% / T: 47.3% (Passed)

BEST1_rs1800009 - Well F2

Entry: BEST1_Ex9_rs1800009

Sample: D14

Position1: C: 3.7% / T: 96.3% (Passed)

BEST1_rs1800009 - Well F5

Entry: BEST1_Ex9_rs1800009

Sample: 1R6

Position1: C: 33.7% / T: 66.3% (Passed)

BEST1_rs1800009 - Well F6

Entry: BEST1_Ex9_rs1800009

Sample: 1R14

Position1: C: 1.5% / T: 98.5% (Passed)

BEST1_rs1800009 - Well F9

Entry: BEST1_Ex9_rs1800009

Sample: 2R6

Position1: C: 33.9% / T: 66.1% (Passed)

BEST1_rs1800009 - Well F10

Entry: BEST1_Ex9_rs1800009

Sample: 2R14

Position1: C: 2.6% / T: 97.4% (Passed)

BEST1_rs1800009 - Well G1

Entry: BEST1_Ex9_rs1800009

Sample: D7

Position1: C: 0.6% / T: 99.4% (Passed)

BEST1_rs1800009 - Well G2

Entry: BEST1_Ex9_rs1800009

Sample: D15

Position1: C: 3.4% / T: 96.6% (Passed)

BEST1_rs1800009 - Well G5

Entry: BEST1_Ex9_rs1800009

Sample: 1R7

Position1: C: 5.7% / T: 94.3% (Passed)

BEST1_rs1800009 - Well G6

Entry: BEST1_Ex9_rs1800009

Sample: 1R15

Position1: C: 2.7% / T: 97.3% (Passed)

BEST1_rs1800009 - Well G9

Entry: BEST1_Ex9_rs1800009

Sample: 2R7

Position1: C: 3.6% / T: 96.4% (Passed)

BEST1_rs1800009 - Well G10

Entry: BEST1_Ex9_rs1800009

Sample: 2R15

Position1: C: 2.2% / T: 97.8% (Passed)

BEST1_rs1800009 - Well H1

Entry: BEST1_Ex9_rs1800009

Sample: D8

Position1: C: 1.9% / T: 98.1% (Passed)

BEST1_rs1800009 - Well H2

Entry: BEST1_Ex9_rs1800009

Sample: D16

Position1: C: 4.9% / T: 95.1% (Passed)

BEST1_rs1800009 - Well H5

Entry: BEST1_Ex9_rs1800009

Sample: 1R8

Position1: C: 3.0% / T: 97.0% (Passed)

BEST1_rs1800009 - Well H6

Entry: BEST1_Ex9_rs1800009

Sample: 1R16

Position1: C: 4.8% / T: 95.2% (Passed)

BEST1_rs1800009 - Well H9

Entry: BEST1_Ex9_rs1800009

Sample: 2R8

Position1: C: 0.3% / T: 99.7% (Passed)

BEST1_rs1800009 - Well H10

Entry: BEST1_Ex9_rs1800009

Sample: 2R16

Position1: C: 2.2% / T: 97.8% (Passed)

*CDHR1*: rs4933980

7.10.14_CDHR1_2_3`(2) - Well A1

Entry: CDHR1-2_3'_rs4933980 (2)

Sample: DNA1

Position1: A: 0.0% / T: 100.0% (Passed)

7.10.14_CDHR1_2_3`(2) - Well A3

Entry: CDHR1-2_3'_rs4933980 (2)

Sample: DNA9

Position1: A: 43.9% / T: 56.1% (Passed)

7.10.14_CDHR1_2_3`(2) - Well A5

Entry: CDHR1-2_3'_rs4933980 (2)

Sample: 1RNA1

Position1: A: 0.0% / T: 100.0% (Failed)

7.10.14_CDHR1_2_3`(2) - Well A7

Entry: CDHR1-2_3'_rs4933980 (2)

Sample: 1RNA9

Position1: A: 31.7% / T: 68.3% (Passed)

7.10.14_CDHR1_2_3`(2) - Well A9

Entry: CDHR1-2_3'_rs4933980 (2)

Sample: 2RNA1

Position1: A: 0.0% / T: 100.0% (Passed)

7.10.14_CDHR1_2_3`(2) - Well A11

Entry: CDHR1-2_3'_rs4933980 (2)

Sample: 2RNA9

Position1: A: 31.4% / T: 68.6% (Passed)

7.10.14_CDHR1_2_3`(2) - Well B1

Entry: CDHR1-2_3'_rs4933980 (2)

Sample: DNA2

Position1: A: 45.3% / T: 54.7% (Passed)

7.10.14_CDHR1_2_3`(2) - Well B3

Entry: CDHR1-2_3'_rs4933980 (2)

Sample: DNA10

Position1: A: 44.1% / T: 55.9% (Passed)

7.10.14_CDHR1_2_3`(2) - Well B5

Entry: CDHR1-2_3'_rs4933980 (2)

Sample: 1RNA2

Position1: A: 30.2% / T: 69.8% (Passed)

7.10.14_CDHR1_2_3`(2) - Well B7

Entry: CDHR1-2_3'_rs4933980 (2)

Sample: 1RNA10

Position1: A: 37.8% / T: 62.2% (Passed)

7.10.14_CDHR1_2_3`(2) - Well B9

Entry: CDHR1-2_3'_rs4933980 (2)

Sample: 2RNA2

Position1: A: 31.8% / T: 68.2% (Passed)

7.10.14_CDHR1_2_3`(2) - Well B11

Entry: CDHR1-2_3'_rs4933980 (2)

Sample: 2RNA10

Position1: A: 36.9% / T: 63.1% (Passed)

7.10.14_CDHR1_2_3`(2) - Well C1

Entry: CDHR1-2_3'_rs4933980 (2)

Sample: DNA3

Position1: A: 42.9% / T: 57.1% (Passed)

7.10.14_CDHR1_2_3`(2) - Well C3

Entry: CDHR1-2_3'_rs4933980 (2)

Sample: DNA11

Position1: A: 46.3% / T: 53.7% (Passed)

7.10.14_CDHR1_2_3`(2) - Well C5

Entry: CDHR1-2_3'_rs4933980 (2)

Sample: 1RNA3

Position1: A: 24.3% / T: 75.7% (Passed)

7.10.14_CDHR1_2_3`(2) - Well C7

Entry: CDHR1-2_3'_rs4933980 (2)

Sample: 1RNA11

Position1: A: 28.7% / T: 71.3% (Passed)

7.10.14_CDHR1_2_3`(2) - Well C9

Entry: CDHR1-2_3'_rs4933980 (2)

Sample: 2RNA3

Position1: A: 23.6% / T: 76.4% (Passed)

7.10.14_CDHR1_2_3`(2) - Well C11

Entry: CDHR1-2_3'_rs4933980 (2)

Sample: 2RNA11

Position1: A: 22.5% / T: 77.5% (Passed)

7.10.14_CDHR1_2_3`(2) - Well D1

Entry: CDHR1-2_3'_rs4933980 (2)

Sample: DNA4

Position1: A: 45.2% / T: 54.8% (Passed)

7.10.14_CDHR1_2_3`(2) - Well D3

Entry: CDHR1-2_3'_rs4933980 (2)

Sample: DNA12

Position1: A: 5.5% / T: 94.5% (Check)

7.10.14_CDHR1_2_3`(2) - Well D5

Entry: CDHR1-2_3'_rs4933980 (2)

Sample: 1RNA4

Position1: A: 31.8% / T: 68.2% (Passed)

7.10.14_CDHR1_2_3`(2) - Well D7

Entry: CDHR1-2_3'_rs4933980 (2)

Sample: 1RNA12

Position1: A: 0.0% / T: 100.0% (Passed)

7.10.14_CDHR1_2_3`(2) - Well D9

Entry: CDHR1-2_3'_rs4933980 (2)

Sample: 2RNA4

Position1: A: 33.7% / T: 66.3% (Passed)

7.10.14_CDHR1_2_3`(2) - Well D11

Entry: CDHR1-2_3'_rs4933980 (2)

Sample: 2RNA12

Position1: A: 0.0% / T: 100.0% (Passed)

7.10.14_CDHR1_2_3`(2) - Well E1

Entry: CDHR1-2_3'_rs4933980 (2)

Sample: DNA5

Position1: A: 99.9% / T: 0.1% (Passed)

7.10.14_CDHR1_2_3`(2) - Well E3

Entry: CDHR1-2_3'_rs4933980 (2)

Sample: DNA13

Position1: A: 47.3% / T: 52.7% (Passed)

7.10.14_CDHR1_2_3`(2) - Well E5

Entry: CDHR1-2_3'_rs4933980 (2)

Sample: 1RNA5

Position1: A: 99.3% / T: 0.7% (Passed)

7.10.14_CDHR1_2_3`(2) - Well E7

Entry: CDHR1-2_3'_rs4933980 (2)

Sample: 1RNA13

Position1: A: 30.9% / T: 69.1% (Passed)

7.10.14_CDHR1_2_3`(2) - Well E9

Entry: CDHR1-2_3'_rs4933980 (2)

Sample: 2RNA5

Position1: A: 96.1% / T: 3.9% (Passed)

7.10.14_CDHR1_2_3`(2) - Well E11

Entry: CDHR1-2_3'_rs4933980 (2)

Sample: 2RNA13

Position1: A: 31.1% / T: 68.9% (Passed)

7.10.14_CDHR1_2_3`(2) - Well F1

Entry: CDHR1-2_3'_rs4933980 (2)

Sample: DNA6

Position1: A: 47.0% / T: 53.0% (Passed)

7.10.14_CDHR1_2_3`(2) - Well F3

Entry: CDHR1-2_3'_rs4933980 (2)

Sample: DNAc+

Position1: A: 0.0% / T: 100.0% (Failed)

7.10.14_CDHR1_2_3`(2) - Well F5

Entry: CDHR1-2_3'_rs4933980 (2)

Sample: 1RNA6

Position1: A: 34.6% / T: 65.4% (Passed)

7.10.14_CDHR1_2_3`(2) - Well F7

Entry: CDHR1-2_3'_rs4933980 (2)

Sample: 1RNAc+

Position1: A: 0.0% / T: 100.0% (Failed)

7.10.14_CDHR1_2_3`(2) - Well F9

Entry: CDHR1-2_3'_rs4933980 (2)

Sample: 2RNA6

Position1: A: 38.8% / T: 61.2% (Passed)

7.10.14_CDHR1_2_3`(2) - Well F11

Entry: CDHR1-2_3'_rs4933980 (2)

Sample: 2RNAc+

Position1: A: 0.0% / T: 100.0% (Failed)

7.10.14_CDHR1_2_3`(2) - Well G1

Entry: CDHR1-2_3'_rs4933980 (2)

Sample: DNA7

Position1: A: 100.0% / T: 0.0% (Passed)

7.10.14_CDHR1_2_3`(2) - Well G3

Entry: CDHR1-2_3'_rs4933980 (2)

Sample: DNAc-

Position1: A: 0.0% / T: 100.0% (Failed)

7.10.14_CDHR1_2_3`(2) - Well G5

Entry: CDHR1-2_3'_rs4933980 (2)

Sample: 1RNA7

Position1: A: 98.6% / T: 1.4% (Passed)

7.10.14_CDHR1_2_3`(2) - Well G7

Entry: CDHR1-2_3'_rs4933980 (2)

Sample: 1RNAc-

Position1: A: 0.0% / T: 100.0% (Failed)

7.10.14_CDHR1_2_3`(2) - Well G9

Entry: CDHR1-2_3'_rs4933980 (2)

Sample: 2RNA7

Position1: A: 100.0% / T: 0.0% (Passed)

7.10.14_CDHR1_2_3`(2) - Well G11

Entry: CDHR1-2_3'_rs4933980 (2)

Sample: 2RNAc-

Position1: A: 0.0% / T: 100.0% (Failed)

7.10.14_CDHR1_2_3`(2) - Well H1

Entry: CDHR1-2_3'_rs4933980 (2)

Sample: DNA8

Position1: A: 0.0% / T: 100.0% (Passed)

7.10.14_CDHR1_2_3`(2) - Well H5

Entry: CDHR1-2_3'_rs4933980 (2)

Sample: 1RNA8

Position1: A: 0.0% / T: 100.0% (Passed)

7.10.14_CDHR1_2_3`(2) - Well H9

Entry: CDHR1-2_3'_rs4933980 (2)

Sample: 2RNA8

Position1: A: 0.0% / T: 100.0% (Passed)

*PROM1*: rs7656732

25.11.15_PROM1_rs7686732_HAS4 - Well B1

Entry: PROM1_rs7686732

Sample: HAS4_DNA1

Position1: C: 55.7% / G: 44.3% (Passed)

25.11.15_PROM1_rs7686732_HAS4 - Well C1

Entry: PROM1_rs7686732

Sample: HAS4_2cDNA2

Position1: C: 60.0% / G: 40.0% (Passed)

25.11.15_PROM1_rs7686732_HAS4 - Well D1

Entry: PROM1_rs7686732

Sample: HAS4_1cDNA1

Position1: C: 61.5% / G: 38.5% (Passed)

**Figure S4.** Pyrograms for: *BEST1* (rs149698, rs180009), *CDHR1* (rs4933980), *PROM1* (rs7656732).

25.11.15_PROM1_rs7686732_HAS4 - Well E1

Entry: PROM1_rs7686732

Sample: HAS4_1cDNA2

Position1: C: 58.6% / G: 41.4% (Passed)

25.11.15_PROM1_rs7686732_HAS4 - Well F1

Entry: PROM1_rs7686732

Sample: HAS4_2cDNA1

Position1: C: 61.3% / G: 38.7% (Passed)

25.11.15_PROM1_rs7686732_HAS4 - Well G1

Entry: PROM1_rs7686732

Sample: HAS4_DNA2

Position1: C: 58.2% / G: 41.8% (Passed)

25.11.15_PROM1_rs7686732_HAS4 - Well H1

Entry: PROM1_rs7686732

Sample: HAS4_-

Position1: C: 0.0% / G: 100.0% (Failed)
