# Supplementary material for: Allelic Expression Imbalance in the Human Retinal Transcriptome and Potential Impact on Inherited Retinal Diseases
Source: Genes (Basel). 2017 Oct 20;8(10):283. doi: 10.3390/genes8100283 (PMC5664133; doi:10.3390/genes8100283)
Supplement: Supplementary file 1 [file genes-08-00283-s001.zip › Table S1. Biological information of collected eye donor samples.docx]

**Table S1.** Biological information of collected eye donor samples for RNA-seq and pyrosequencing. RNA quality is indicated by the RIN (ranging from 1 to 10, 10 being the maximum quality). RNA-seq was carried out according to the guidelines of Microarray Facility Tuebingen Services. HAS4 and 13 were used to develop the AEI pipeline.

| **Sample** | **Gender** | **Eyes received** | **Age** | **RIN** | **RNA-seq** | **Reads (QC-passed)** | **Mapped reads** | **AEI pipeline reads (QC-passed)** | **AEI pipeline Mapped reads (STAR2-pass)** |
| --- | --- | --- | --- | --- | --- | --- | --- | --- | --- |
| HAS1 | ♂ |  |  | 2.5 |  |  |  |  |  |
| HAS2 | ♀ |  | 1968 | 2.8 |  |  |  |  |  |
| HAS3 | ♂ | 72 h post mortem | 1943 | n.a. |  |  |  |  |  |
| HAS4 | ♂ | 24 h post mortem | 1951 | 7 | Yes | 78,422,345 | 48,695,477 | 91,223,997 | 74,586,208 |
| HAS5 | ♂ | 48 h post mortem | 1950 | 4.4 |  |  |  |  |  |
| HAS6 |  | 15 h post mortem | 1948 | 6.5 |  |  |  |  |  |
| HAS7 | ♂ | 80 h post mortem | 1928 | 4.8 |  |  |  |  |  |
| HAS8 | ♂ |  | 1935 | 5.9 |  |  |  |  |  |
| HAS9 | ♂ | 30 h post mortem | 1935 | 6.8 |  |  |  |  |  |
| HAS10 | ♂ | 5 h post mortem | 1946 | 7.4 |  |  |  |  |  |
| HAS11 | ♂ | 80 h post mortem | 1930 | 3.3 |  |  |  |  |  |
| HAS12 | ♂ | 8 h post mortem | 1925 | 7.6 |  |  |  |  |  |
| HAS13 | ♂ |  |  | 6.7 | Yes | 65,162,182 | 41,521,344 | 88,877,421 | 71,032,876 |
| HAS14 | ♂ | 14 h post mortem |  |  |  |  |  |  |  |
| HAS15 | ♀ | 8 h post mortem |  |  |  |  |  |  |  |
| HAS16 | ♂ | 7 h post mortem |  |  |  |  |  |  |  |
| HAS17 | ♀ | 13 h post mortem |  |  |  |  |  |  |  |

HAS – human eye donor; AEI – allelic expression imbalance; RIN – RNA integrity number; QC – quality control; n.a not analyzed
