# Supplementary material for: Allelic Expression Imbalance in the Human Retinal Transcriptome and Potential Impact on Inherited Retinal Diseases
Source: Genes (Basel). 2017 Oct 20;8(10):283. doi: 10.3390/genes8100283 (PMC5664133; doi:10.3390/genes8100283)
Supplement: Supplementary file 1 [file genes-08-00283-s001.zip › Table S2. List of primers.docx]

**Table S2.** List of pyrosequencing, PCR and/or Sanger sequencing primers. Score of pyrosequencing assay is also provided

| **Assay** | **Type** | **Forward 5'-3'** | **Reverse 5'-3'** | **Sequencing 5'-3'** | **Score** |
| --- | --- | --- | --- | --- | --- |
| *ABCA4*_rs1762114 | Pyrosequencing | TGGGCTACTGTCCTCAGTTTG | (bio)AAGCCGGGCATAAAGGTAA | CCTCAGTTTGATGCAAT | 91 |
|  | PCR-Sanger | TCAAAATATGGGCTACTGTCCTCA | GTGGCCACAACAAAACATTTTTCA |  |  |
| *ABHD12*_rs6107027 | Pyrosequencing | (bio)ACGCCTCCAGATGCCCTTATATT | CTGAAAATGGATGGCTCTTAGCTT | GGATGGCTCTTAGCTTCT | 98 |
|  | PCR-Sanger | TCCTGCCTACCTCTCAGTTCC | ACTTGCCACTGAAAATGGATGG |  |  |
| *BBS5*_rs7589199 | Pyrosequencing | n.a |  |  |  |
|  | PCR-Sanger |  |  |  |  |
| **BEST1*_rs149698 | Pyrosequencing | (bio)TGTGGACGCCTTCAAGTCTG | GGGGGAAGAACATGGGAGTG | GGGAGTGGGGCTGAG | 100 |
|  | PCR-Sanger | AACAGAACGTTAGGGGCCAG | TTCTGGGTGCTCCATCAAGG |  |  |
| **BEST1*_1800009 | Pyrosequencing | AGCGATGGGGCCTTGATG | (bio)GGGGATCTCTGGCATATCCG | TCAAGTGAGGAGGAAAA | 91 |
|  | PCR-Sanger | TGGGGCCAAGAAAAGTTTTGA | GGGGATCTCTGGCATATCCG |  |  |
| *C3*_rs17030 | Pyrosequencing | ACACTTGGGTGGAGCACTGG | (bio)CCGAGGTCCTGGCATTGTTT | GGGTGGAGCACTGGC | 91 |
|  | PCR-Sanger |  |  |  |  |
| *CC2D2A*_rs4698387 | Pyrosequencing |  |  |  |  |
|  | PCR-Sanger |  |  |  |  |
| *CDHR1*_rs4244947 | Pyrosequencing | n.a |  |  |  |
|  | PCR-Sanger |  |  |  |  |
| *CDHR1*_rs4933980 | Pyrosequencing | TCTCATCCTGACCCCTCTGTCT | (bio)TGGGCTACCATGAAGGTGAGAGT | AGCTTCTTTTGCTTGG | 91 |
|  | PCR-Sanger | AACTTCCCTGGTACAACAAAGTC | GTGGTCTACGGATCTTGAGGG |  |  |
| *CDHR1*_rs10509491 | Pyrosequencing | n.a |  |  |  |
|  | PCR-Sanger |  |  |  |  |
| *CDHR1*_rs7895270 | Pyrosequencing | n.a |  |  |  |
|  | PCR-Sanger |  |  |  |  |
| *CDHR1*_rs2279229 | Pyrosequencing | n.a |  |  |  |
|  | PCR-Sanger |  |  |  |  |
| *CNGB1*_rs17821448 | Pyrosequencing | GGGTACTGACCTGGCTCATGAA | (bio)GTCCTCCGTGATGCTGTGAAC | GACCTGGCTCATGAA | 100 |
|  | PCR-Sanger | GCTAGGGGAAGTTGAGGGC | TCCTCCGTGATGCTGTGAAC |  |  |
| **Assay** | **Type** | **Forward 5'-3'** | **Reverse 5'-3'** | **Sequencing 5'-3'** | **Score** |
| **COL11A1*_rs2229783 | Pyrosequencing | TCCCTCAATTCCCTGAAACAA | (bio)TTTACAAGTTCGGGCTGGATT | TCCCTGAAACAAGACAT | 99 |
|  | PCR-Sanger | GGTCCACCTGGTGAAGTCAT | GATGGCTGAGTTGCAGGTCT |  |  |
| *FLVCR1*_rs10864027 | Pyrosequencing | (bio)TTACTCATGGTGAAAGGCTAAGTG | TCCTTGCAGTAATGAGTGAGTTTT | CCTCCCTGCTCTCTT | 89 |
|  | PCR-Sanger |  |  |  |  |
| *GRK1*_rs9796035 | Pyrosequencing | poor assay. Score 69 |  |  |  |
|  | PCR-Sanger |  |  |  |  |
| *GRM6*_rs11746675 | Pyrosequencing | AGGAACCCCTGTGATGTTCAAC | (bio)GTCGCCTGGTACTGGAAGATGTC | TGTTCAACGAGAACGG | 90 |
|  | PCR-Sanger | GACCACCGAGGATGACGGA | TCCAGTCTGAGGGTCTCTGCC |  |  |
| *GRM6*_rs2067011 | Pyrosequencing | n.a |  |  |  |
|  | PCR-Sanger |  |  |  |  |
| *GRM6*_rs2071246 | Pyrosequencing | n.a |  |  |  |
|  | PCR-Sanger |  |  |  |  |
| *IDH3B*_rs5026920 | Pyrosequencing | n.a |  |  |  |
|  | PCR-Sanger |  |  |  |  |
| *INPP5E*_rs1128874 | Pyrosequencing | exon edge |  |  |  |
|  | PCR-Sanger |  |  |  |  |
| *INPP5E*_rs10870194 | Pyrosequencing |  |  |  |  |
|  | PCR-Sanger |  |  |  |  |
| *INPP5E*_rs35763810 | Pyrosequencing | exon edge |  |  |  |
|  | PCR-Sanger |  |  |  |  |
| *MYO7A*_rs2276288 | Pyrosequencing | CCAAGCAGCGTGGGGACT | (bio)GCGGTGGCATGGTGACAG | GACTTCCCCACCGAC | 93 |
|  | PCR-Sanger | TGCCAAGGGAGACCTCATCA | CCAGGCCACATACCACAATCT |  |  |
| *PRCD*_rs5742903 | Pyrosequencing | n.a |  |  |  |
|  | PCR-Sanger |  |  |  |  |
| *PRCD*_rs895157 | Pyrosequencing | n.a |  |  |  |
|  | PCR-Sanger |  |  |  |  |
| **PROM1*_rs3130 | Pyrosequencing | poor assay. Score 80 |  |  |  |
|  | PCR-Sanger |  |  |  |  |
| **Assay** | **Type** | **Forward 5'-3'** | **Reverse 5'-3'** | **Sequencing 5'-3'** | **Score** |
| (**PROM1_*rs7686732) | Pyrosequencing | (bio)TTCGTTCAAAAGTGGAGTTGTT | CATGTTAGCTGCACTCCAATTAAT | CACTCCAATTAATGTTTATC | 90 |
|  | PCR-Sanger | TGCTGTTCGTTCAAAAGTGGAG | CATCCATGCTGGACACCAGA |  |  |
| **PRPH2*_rs425876 | Pyrosequencing | (bio)ACCCCGAGGAATCTGAGAG | TTGCCCAGCTTCTTCACACTC | CAGGTCTCCGGCACG | 89 |
|  | PCR-Sanger | GTGACCATTACAATTGGGCTGC | CCCAGCTTCTTCACACTCTCC |  |  |
| **RP1*_rs61739567 | Pyrosequencing | (bio)TTCTTGGGGTTAGAGGAAGAAGGT | CCCACAACTAACAATGATGTGTGC | TGTGTGCAAGAAATTCAT | 91 |
|  | PCR-Sanger | CAGTAACACATTTGACTTGATGGGT | TCTTGAGCCCTGGAATCTGT |  |  |
| *WFS1*_rs1046319 | Pyrosequencing | n.a |  |  |  |
|  | PCR-Sanger |  |  |  |  |
