# Supplementary material for: Allelic Expression Imbalance in the Human Retinal Transcriptome and Potential Impact on Inherited Retinal Diseases
Source: Genes (Basel). 2017 Oct 20;8(10):283. doi: 10.3390/genes8100283 (PMC5664133; doi:10.3390/genes8100283)
Supplement: Supplementary file 1 [file genes-08-00283-s001.zip › Table S3. RNA-seq AEI SNP list.docx]

**Table S3.** RNA-seq AEI SNP list. Heterozygosity was checked by Sanger sequencing of PCR amplified genomic DNA from 17 independent sclera donors. Pyrosequencing assay score was considered for assay selection (assays with scores lower than 90 were rejected). In column five we show the number of samples that were found to be imbalanced at RNA level with balanced alleles at DNA level. Integrative Genomics Viewer (IGV) SNP visual assessment was carried out by using two sorts of .BAM files per sample (data cleanup and no cleanup).

| **ID** | **Symbol** | **Sanger heterozygosity screening in 17 samples** | **Pyro-sequencing assay score** | **Pyrosequencing RNA/DNA positive** | **RNA-seq visual inspection (positive AEI)** | **Comments** |
| --- | --- | --- | --- | --- | --- | --- |
| rs1762114 | *ABCA4* | 5 | 98 | 0 |  |  |
| rs6107027 | *ABHD12* | 5 | 96 | 0 |  | Unstable assay |
| rs7589199 | *BBS5* |  |  |  |  |  |
| rs149698 | *BEST1* | 6 |  | 2 (HAS6,13) | HAS13 |  |
| rs1800009 | *BEST1* | 5 |  | 2 (HAS6,13) | HAS13 |  |
| rs17030 | *C3* | 9 |  |  |  |  |
| rs4698387 | *CC2D2A* |  |  |  |  |  |
| rs4244947 | *CDHR1* |  |  |  |  |  |
| rs4933980 | *CDHR1* | 8 | 91 | 8 (HAS2,3,  4,6,9,10,11,13) | |  |
| rs10509491 | *CDHR1* |  |  |  |  |  |
| rs7895270 | *CDHR1* |  |  |  |  |  |
| rs2279229 | *CDHR1* |  |  |  |  |  |
| rs17821448 | *CNGB1* | 13 | 100 | 0 |  |  |
| rs2229783 | *COL11A1* | 4 | 99 | 0 |  |  |
| rs10864027 | *FLVCR1* |  | 89 |  |  |  |
| rs9796035 | *GRK1* | 8 | 69 |  | HAS4 |  |
| rs11746675 | *GRM6* |  | 90 |  |  | Unstable assay |
| rs2067011 | *GRM6* |  |  |  |  |  |
| rs2071246 | *GRM6* | 8 | 100 | 0 |  |  |
| rs5026920 | *IDH3B* |  |  |  |  |  |
| rs33982662 | *INPP5E* |  |  |  | HAS13 | No pyrosequencing assay was possible either due to poor performance or close to exon edge |
| rs1128874 | *INPP5E* |  |  |  |  |  |
| rs10870194 | *INPP5E* |  |  |  | HAS13 |  |
| rs2276288 | *MYO7A* | 7 | 93 |  |  | Unstable assay |
| rs895157 | *PRCD* |  |  |  |  |  |
| rs5742903 | *PRCD* |  |  |  |  |  |
| rs3130 | *PROM1* |  | 80 |  |  | rs2240688 was analyzed instead: HAS6 positive |
| rs22406088 | *PROM1* |  | 91 | 1 |  |  |
| rs425876 | *PRPH2* | 4 | 89 | 0 |  |  |
| rs61739567 | *RP1* | 7 | 91 | 0 |  |  |
| rs1046319 | *WFS1* |  |  |  |  | Double SNP |
