# Supplementary material for: Allelic Expression Imbalance in the Human Retinal Transcriptome and Potential Impact on Inherited Retinal Diseases
Source: Genes (Basel). 2017 Oct 20;8(10):283. doi: 10.3390/genes8100283 (PMC5664133; doi:10.3390/genes8100283)
Supplement: Supplementary file 1 [file genes-08-00283-s001.zip › Table S4. Relative allelic percentage.docx]

**Table S4.** Relative allelic percentage in inherited retinal diseases (IRD) genes displaying allelic expression imbalance (AEI) frequencies higher than 10% in our 52 RNA-seq data set. Highlighted with an asterisk are those genes known to be associated to autosomal dominant inheritance.

| **Symbol** | **Samples in top mean±2*SD ratios** | **Heterozygous samples** | **Allelic ratio  < 0.66** | **Allelic ratio  > 1.5** | **Sum imbalanced samples** | **AEI frequency in heterozygous samples** | **AEI frequency in 52 samples** | **% Allele A** | **% Allele B** | **SEM % Allele A** |
| --- | --- | --- | --- | --- | --- | --- | --- | --- | --- | --- |
| *ABCA4*_rs1762114 | 7 | 16 | 0 | 8 | 8 | 50.0% | 15.4% | 6.3% | 93.7% | 4.4% |
| *ABHD12*_rs6107027 | 1 | 20 | 11 | 1 | 12 | 60.0% | 23.1% | 33.8% | 66.2% | 3.0% |
| *BBS5*_rs7589199 | 3 | 11 | 5 | 3 | 8 | 72.7% | 15.4% | 22.9% | 77.1% | 6.3% |
| **BEST1*_rs149698 | 4 | 22 | 1 | 6 | 7 | 31.8% | 13.5% | 23.1% | 76.9% | 2.6% |
| **BEST1*_1800009 | 2 | 20 | 4 | 2 | 6 | 30.0% | 11.5% | 27.6% | 72.4% | 5.0% |
| *C3*_rs17030 | 2 | 29 | 2 | 6 | 8 | 27.6% | 15.4% | 25.4% | 74.6% | 5.5% |
| *CC2D2A*_rs4698387 | 1 | 17 | 11 | 0 | 11 | 64.7% | 21.2% | 33.9% | 66.1% | 1.5% |
| *CDHR1*_rs4244947 | 16 | 23 | 21 | 2 | 23 | 100.0% | 44.2% | 19.3% | 80.7% | 1.4% |
| *CDHR1*_rs4933980 | 17 | 21 | 21 | 0 | 21 | 100.0% | 40.4% | 20.4% | 79.6% | 1.0% |
| *CDHR1*_rs10509491 | 1 | 20 | 11 | 0 | 11 | 55.0% | 21.2% | 33.4% | 66.6% | 1.8% |
| *CDHR1*_rs7895270 | 6 | 16 | 10 | 0 | 10 | 62.5% | 19.2% | 23.0% | 77.0% | 2.0% |
| *CDHR1*_rs2279229 | 1 | 15 | 7 | 1 | 8 | 53.3% | 15.4% | 33.5% | 66.5% | 4.6% |
| *CNGB1*_rs17821448 | 10 | 30 | 1 | 10 | 11 | 36.7% | 21.2% | 6.6% | 93.4% | 5.5% |
| **COL11A1*_rs2229783 | 2 | 19 | 2 | 7 | 9 | 47.4% | 17.3% | 33.1% | 66.9% | 3.0% |
| *FLVCR1*_rs10864027 | 1 | 24 | 5 | 1 | 6 | 25.0% | 11.5% | 29.4% | 70.6% | 5.6% |
| *GRK1*_rs9796035 | 3 | 21 | 10 | 2 | 12 | 57.1% | 23.1% | 29.5% | 70.5% | 3.3% |
| *GRM6*_rs11746675 | 2 | 32 | 5 | 4 | 9 | 28.1% | 17.3% | 30.4% | 69.6% | 4.4% |
| *GRM6*_rs2067011 | 1 | 29 | 4 | 3 | 7 | 24.1% | 13.5% | 29.3% | 70.7% | 5.3% |
| *GRM6*_rs2071246 | 3 | 22 | 2 | 4 | 6 | 27.3% | 11.5% | 22.2% | 77.8% | 7.4% |
| *IDH3B*_rs5026920 | 1 | 12 | 8 | 0 | 8 | 66.7% | 15.4% | 32.1% | 67.9% | 2.2% |
| *INPP5E*_rs1128874 | 2 | 27 | 8 | 1 | 9 | 33.3% | 17.3% | 32.1% | 67.9% | 3.7% |
| *INPP5E*_rs10870194 | 2 | 24 | 0 | 24 | 24 | 100.0% | 46.2% | 8.0% | 92.0% | 1.2% |
| *INPP5E*_rs35763810 | 3 | 20 | 0 | 6 | 6 | 30.0% | 11.5% | 31.9% | 68.1% | 6.0% |
| *MYO7A*_rs2276288 | 3 | 17 | 13 | 1 | 14 | 82.4% | 26.9% | 33.3% | 66.7% | 2.5% |
| *PRCD*_rs5742903 | 1 | 25 | 7 | 2 | 9 | 36.0% | 17.3% | 28.9% | 71.1% | 5.2% |
| *PRCD*_rs895157 | 1 | 17 | 6 | 1 | 7 | 41.2% | 13.5% | 31.0% | 69.0% | 4.2% |
| **PROM1*_rs3130 | 8 | 20 | 0 | 19 | 19 | 95.0% | 36.5% | 27.8% | 72.2% | 1.9% |
| **PRPH2*_rs425876 | 6 | 14 | 1 | 7 | 8 | 57.1% | 15.4% | 33.9% | 66.1% | 2.8% |
| **RP1*_rs61739567 | 2 | 11 | 0 | 8 | 8 | 72.7% | 15.4% | 28.8% | 71.2% | 2.6% |
| *WFS1*_rs1046319 | 1 | 12 | 0 | 7 | 7 | 58.3% | 13.5% | 9.1% | 90.9% | 3.6% |
