# Supplementary material for: Allelic Expression Imbalance in the Human Retinal Transcriptome and Potential Impact on Inherited Retinal Diseases
Source: Genes (Basel). 2017 Oct 20;8(10):283. doi: 10.3390/genes8100283 (PMC5664133; doi:10.3390/genes8100283)
Supplement: Supplementary file 1 [file genes-08-00283-s001.zip › Table S5. List of SNPs in LD with AEI candidate SNPs.docx]

**Table S5.** List of SNPs in linkage disequilibrium (LD) with allelic expression imbalance (AEI) candidate SNPs belonging to autosomal dominant acting inherited retinal diseases IRD genes found during the RNA-seq analysis. Highlighted in red are the SNPs found to be imbalanced in the RNA-seq. Regulatory features were obtained in HaploReg ( <http://archive.broadinstitute.org/mammals/haploreg/haploreg.php>) (for further details make the query with SNPs listed in Table 1 in HaploReg v4.1).

|  | **Chr** | **Pos (hg38)** | **LD (r²)** | **LD (D')** | **Variant** | **Ref** | **Alt** | **EUR frequency** | **Promoter histone marks** | **Enhancer histone marks** | **DNAse** | **Proteins bound** | **Motifs changed** | **GRASP QTL hits** | **Selected eQTL hits** | **dbSNP functional annotation** |
| --- | --- | --- | --- | --- | --- | --- | --- | --- | --- | --- | --- | --- | --- | --- | --- | --- |
|  | 11 | 61962564 | 1 | 1 | rs149698 | G | A | 0.31 | 1 tissue | 16 tissues | 20 tissues | 1 bound protein | 6 altered motifs |  | 1 hit | synonymous |
| *BEST1* | 11 | 61972114 | 0.83 | 0.96 | rs3094402 | A | C | 0.33 | 5 tissues | 23 tissues | 52 tissues | 15 bound proteins | 1 altered motif |  |  |  |
|  | 11 | 61975494 | 0.84 | 0.97 | rs195446 | C | T | 0.33 | 3 tissues | 18 tissues | 12 tissues | 5 bound proteins | 4 altered motifs |  | 2 hits |  |
|  | 4 | 15966951 | 0.99 | 1 | rs4698434 | T | C | 0.54 |  |  |  |  | 1 altered motif | 1 hit | 1 hit |  |
| *PROM1* | 4 | 15968315 | 1 | 1 | rs3130 | T | C | 0.54 |  |  |  |  | 7 altered motifs |  | 1 hit | 3'-UTR |
|  | 4 | 15972351 | 0.96 | 0.99 | rs4698130 | A | G | 0.53 |  |  |  |  | 2 altered motifs |  | 1 hit | intronic |
|  | 6 | 42628496 | 0.8 | -0.89 | rs62414627 | A | T | 0.11 |  | 2 tissues |  |  | 1 altered motif |  |  | intronic |
| *PRPH2* | 6 | 42640033 | 0.8 | 0.89 | rs2747119 | T | A | 0.89 |  |  |  |  | 14 altered motifs |  |  | intronic |
|  | 6 | 42644574 | 0.86 | 0.96 | rs7767888 | C | T | 0.88 |  |  |  |  | 11 altered motifs |  |  | intronic |
|  | **Chr** | **Pos (hg38)** | **LD (r²)** | **LD (D')** | **Variant** | **Ref** | **Alt** | **EUR frequency** | **Promoter histone marks** | **Enhancer histone marks** | **DNAse** | **Proteins bound** | **Motifs changed** | **GRASP QTL hits** | **Selected eQTL hits** | **dbSNP functional annotation** |
|  | 6 | 42645237 | 0.86 | 0.96 | rs9367159 | G | A | 0.88 |  |  |  |  |  |  |  | intronic |
|  | 6 | 42648488 | 0.86 | 0.96 | rs1040611 | T | C | 0.88 |  |  |  |  | 6 altered motifs |  |  | intronic |
|  | 6 | 42649682 | 0.86 | 0.96 | rs9381205 | A | G | 0.88 |  |  |  |  | 5 altered motifs |  |  | intronic |
|  | 6 | 42651652 | 0.89 | 0.97 | rs2057128 | A | G | 0.88 |  |  |  |  | 5 altered motifs |  |  | intronic |
|  | 6 | 42662841 | 0.96 | 0.99 | rs761538 | A | G | 0.89 |  |  |  |  | 2 altered motifs |  |  | intronic |
|  | 6 | 42664387 | 1 | 1 | rs2395923 | G | A | 0.89 |  | 1 tissue |  |  | 2 altered motifs |  |  | intronic |
| *PRPH2* | 6 | 42668092 | 1 | 1 | rs7768961 | C | A | 0.89 |  |  |  | 1 bound protein | 4 altered motifs |  |  | intronic |
|  | 6 | 42673434 | 0.83 | 1 | rs2013698 | T | G | 0.87 |  |  |  |  | 4 altered motifs |  |  | intronic |
|  | 6 | 42676324 | 1 | 1 | rs2144083 | A | G | 0.89 |  | 1 tissue |  |  | 2 altered motifs |  |  | intronic |
|  | 6 | 42678780 | 1 | 1 | rs2295153 | T | A | 0.89 |  |  |  |  | 9 altered motifs | 1 hit |  | intronic |
|  | 6 | 42683565 | 1 | 1 | rs399013 | A | G | 0.89 |  |  |  |  | 3 altered motifs | 1 hit |  | intronic |
|  | 6 | 42686567 | 0.99 | 1 | rs659154 | C | T | 0.89 |  |  |  |  |  |  |  | intronic |
|  | 6 | 42687981 | 0.81 | 1 | rs60340732 | AT | A | 0.87 |  |  |  |  | 4 altered motifs |  |  | intronic |
|  | **Chr** | **Pos (hg38)** | **LD (r²)** | **LD (D')** | **Variant** | **Ref** | **Alt** | **EUR frequency** | **Promoter histone marks** | **Enhancer histone marks** | **DNAse** | **Proteins bound** | **Motifs changed** | **GRASP QTL hits** | **Selected eQTL hits** | **dbSNP functional annotation** |
|  | 6 | 42690792 | 1 | 1 | rs374036 | T | C | 0.89 |  | 10 tissues |  |  | 2 altered motifs |  |  | intronic |
|  | 6 | 42695333 | 0.91 | 1 | rs448141 | T | C | 0.88 | 1 tissue | 9 tissues | 1 tissue |  | 1 altered motif |  |  |  |
|  | 6 | 42695549 | 1 | 1 | rs621627 | C | T | 0.89 |  | 6 tissues |  |  | 1 altered motif |  |  |  |
| *PRPH2* | 6 | 42695756 | 0.82 | 1 | rs450579 | A | C | 0.87 |  | 5 tissues |  |  | 4 altered motifs |  | 1 hit |  |
|  | 6 | 42698407 | 1 | 1 | rs425876 | C | T | 0.89 |  | 1 tissue | 1 tissue | 1 bound protein | 4 altered motifs |  |  | missense |
|  | 6 | 42698876 | 0.96 | 0.99 | rs665442 | G | T | 0.89 |  |  |  |  | 2 altered motifs |  |  | intronic |
|  | 6 | 42700064 | 0.99 | 1 | rs377118 | G | T | 0.89 |  | 1 tissue |  |  | 1 altered motif |  |  | intronic |
|  | 8 | 54612471 | 0.93 | 0.98 | rs4327841 | A | G | 0.44 |  |  |  |  |  |  |  |  |
| *RP1* | 8 | 54624325 | 1 | 1 | rs62514616 | G | A | 0.43 |  | 1 tissue |  |  | 1 altered motif |  |  | intronic |
|  | 8 | 54626835 | 1 | 1 | rs2293869 | A | T | 0.43 |  | 3 tissues |  |  | 6 altered motifs |  |  | missense |
|  | **Chr** | **Pos (hg38)** | **LD (r²)** | **LD (D')** | **Variant** | **Ref** | **Alt** | **EUR frequency** | **Promoter histone marks** | **Enhancer histone marks** | **DNAse** | **Proteins bound** | **Motifs changed** | **GRASP QTL hits** | **Selected eQTL hits** | **dbSNP functional annotation** |
|  | 8 | 54629980 | 1 | 1 | rs61739567 | G | A | 0.43 |  | 6 tissues | 1 tissue |  | 1 altered motif |  |  | missense |
|  | 8 | 54634506 | 1 | 1 | rs11300666 | GC | G | 0.43 |  |  |  |  | 6 altered motifs |  |  |  |
|  | 8 | 54637376 | 1 | 1 | rs62514617 | G | T | 0.43 |  |  |  |  | 2 altered motifs |  |  |  |
|  | 8 | 54639577 | 1 | 1 | rs62514618 | T | C | 0.43 |  |  |  |  | 3 altered motifs |  |  |  |
|  | 8 | 54639750 | 1 | 1 | rs7001303 | A | G | 0.43 |  |  |  |  | 1 altered motif | 1 hit |  |  |
| *RP1* | 8 | 54646146 | 1 | 1 | rs17318285 | A | T | 0.43 |  |  |  |  | 1 altered motif |  |  |  |
|  | 8 | 54649822 | 1 | 1 | rs17403182 | A | G | 0.43 |  |  |  | 1 bound protein | 1 altered motif |  |  |  |
|  | 8 | 54658740 | 0.97 | 1 | rs72650333 | G | A | 0.42 |  |  |  |  |  |  |  |  |
|  | 8 | 54665479 | 0.95 | 0.99 | rs4606036 | G | A | 0.42 |  |  |  |  | 2 altered motifs |  |  |  |
|  | 8 | 54668653 | 0.94 | 0.98 | rs80245998 | G | A | 0.43 |  |  |  |  | 3 altered motifs |  |  |  |
|  | 8 | 54670459 | 0.95 | 0.99 | rs7462132 | G | A | 0.42 |  |  |  |  | 1 altered motif |  |  |  |
|  | **Chr** | **Pos (hg38)** | **LD (r²)** | **LD (D')** | **Variant** | **Ref** | **Alt** | **EUR frequency** | **Promoter histone marks** | **Enhancer histone marks** | **DNAse** | **Proteins bound** | **Motifs changed** | **GRASP QTL hits** | **Selected eQTL hits** | **dbSNP functional annotation** |
|  | 8 | 54671308 | 0.9 | 0.96 | rs7831784 | G | A | 0.43 |  |  |  |  | 4 altered motifs |  |  |  |
|  | 8 | 54672159 | 0.9 | 0.96 | rs62514622 | C | T | 0.42 |  |  |  |  | 1 altered motif |  |  |  |
|  | 8 | 54673079 | 0.9 | 0.96 | rs41444144 | C | T | 0.42 |  |  |  |  | 1 altered motif |  |  |  |
|  | 8 | 54674732 | 0.9 | 0.96 | rs146945978 | GA | G | 0.42 |  |  |  |  | 4 altered motifs |  |  |  |
|  | 8 | 54677731 | 0.9 | 0.96 | rs56168676 | C | T | 0.42 |  |  |  |  | 9 altered motifs |  |  |  |
|  | 8 | 54681026 | 0.89 | 0.95 | rs9774102 | C | T | 0.43 |  |  |  |  | 3 altered motifs |  |  |  |
| *RP1* | 8 | 54686038 | 0.9 | 0.96 | rs72650340 | T | C | 0.42 |  |  |  |  | 3 altered motifs |  |  |  |
|  | 8 | 54688341 | 0.89 | 0.96 | rs145017125 | C | T | 0.42 |  |  |  |  | 5 altered motifs |  |  |  |
|  | 8 | 54690347 | 0.9 | 0.96 | rs9650311 | A | T | 0.42 |  | 1 tissue | 1 tissue |  |  |  |  |  |
|  | 8 | 54691457 | 0.9 | 0.96 | rs7015807 | C | T | 0.42 |  | 1 tissue | 2 tissues |  | 3 altered motifs |  |  |  |
|  | 8 | 54697391 | 0.89 | 0.96 | rs62514627 | C | T | 0.42 |  |  |  |  | 3 altered motifs |  |  |  |
|  | 8 | 54704679 | 0.89 | 0.96 | rs62514628 | G | A | 0.42 |  |  |  |  | 5 altered motifs |  |  | intronic |
|  | **Chr** | **Pos (hg38)** | **LD (r²)** | **LD (D')** | **Variant** | **Ref** | **Alt** | **EUR frequency** | **Promoter histone marks** | **Enhancer histone marks** | **DNAse** | **Proteins bound** | **Motifs changed** | **GRASP QTL hits** | **Selected eQTL hits** | **dbSNP functional annotation** |
|  | 8 | 54705008 | 0.89 | 0.96 | rs62514629 | C | T | 0.42 |  |  |  |  | 4 altered motifs |  |  | intronic |
|  | 8 | 54709457 | 0.87 | 0.96 | rs17319102 | C | A | 0.42 |  | 2 tissues | 1 tissue |  | 5 altered motifs |  |  | intronic |
|  | 8 | 54710210 | 0.89 | 0.96 | rs7815076 | G | T | 0.42 |  | 1 tissue | 1 tissue |  | 2 altered motifs |  |  | intronic |
|  | 8 | 54718409 | 0.89 | 0.96 | rs56094449 | C | G | 0.42 |  |  |  |  | 3 altered motifs |  |  | intronic |
|  | 8 | 54725331 | 0.84 | 0.94 | rs17319305 | C | T | 0.42 |  |  |  |  | 2 altered motifs |  |  | intronic |
| *RP1* | 8 | 54725893 | 0.83 | 0.94 | rs17319332 | A | G | 0.42 |  | 2 tissues |  |  |  |  |  | intronic |
|  | 8 | 54740019 | 0.84 | 0.94 | rs11778137 | G | A | 0.42 |  |  |  |  | 3 altered motifs |  |  | intronic |
|  | 8 | 54744233 | 0.84 | 0.94 | rs55642754 | G | T | 0.42 |  | 3 tissues | 6 tissues | 1 bound protein | 2 altered motifs |  |  | intronic |
|  | 8 | 54744499 | 0.84 | 0.94 | rs62514651 | C | T | 0.42 |  | 2 tissues | 2 tissues |  | 1 altered motif |  |  | intronic |
|  | 8 | 54763915 | 0.8 | 0.92 | rs10958426 | T | G | 0.42 |  |  |  |  | 5 altered motifs |  |  | intronic |
|  | 8 | 54764129 | 0.8 | 0.92 | rs10958427 | G | A | 0.42 |  |  | 4 tissues |  | 1 altered motif |  |  | intronic |
|  | 8 | 54770606 | 0.8 | 0.92 | rs17320233 | A | G | 0.42 |  | 1 tissue |  |  | 7 altered motifs |  |  | intronic |

GRASP: Genome-Wide Repository of Associations Between SNPs and Phenotypes; LD: Linkage desequilibrium
